# Artemin is hypoxia responsive and promotes oncogenicity and increased tumor initiating capacity in hepatocellular carcinoma

**DOI:** 10.18632/oncotarget.6572

**Published:** 2015-12-12

**Authors:** Min Zhang, Weijie Zhang, Zhengsheng Wu, Shumin Liu, Linchong Sun, Yanghao Zhong, Xiao Zhang, Xiangjun Kong, Pengxu Qian, Huafeng Zhang, Peter E. Lobie, Tao Zhu

**Affiliations:** ^1^ The CAS Key Laboratory of Innate Immunity and Chronic Disease, School of Life Sciences and Medical Center, University of Science and Technology of China, Hefei, China; ^2^ Hefei National Laboratory for Physical Sciences at Microscale, Hefei, China; ^3^ Department of Pathology, Anhui Medical University, Hefei, China; ^4^ Cancer Science Institute of Singapore and Department of Pharmacology, National University of Singapore, Singapore

**Keywords:** hypoxia, ARTN, hepatocellular carcinoma, cancer stem cell

## Abstract

Hypoxia has been reported to regulate the cancer stem cell (CSC) population yet the underlying mechanism is poorly characterized. Herein, we show that Artemin (ARTN), a member of the glial cell derived neurotrophic factor family of ligands, is a hypoxia-responsive factor and is essential for hypoxia-induced CSC expansion in hepatocellular carcinoma (HCC). Clinically, elevated expression of ARTN in HCC was associated with larger tumor size, faster relapse and shorter survival. *In vitro*, HCC cells with forced expression of ARTN exhibited reduced apoptosis, increased proliferation, epithelial-mesenchymal transition (EMT) and enhanced motility. Additionally, ARTN dramatically increased xenograft tumor size and metastasis *in vivo*. Moreover, ARTN also enhanced tumorsphere formation and the tumor initiating capacity of HCC cells, consequent to expansion of the CD133^+^ CSC population. ARTN transcription was directly activated by hypoxia-induced factor-1α (HIF-1α) and hypoxia induced ARTN promoted EMT and increased the CSC population via AKT signaling. We herein identify a novel HIF-1α/ARTN axis promoting CSC-like behavior in hypoxic environments which implicates ARTN as a valuable therapeutic target for HCC.

## INTRODUCTION

Hepatocellular carcinoma (HCC), the most prevalent primary malignancy of the liver, has become the second leading cause of cancer death globally. Furthermore, the incidence of HCC has been rising in the past decade [1]. The prevalence of HCC in parts of Africa and Asia is predominantly due to chronic hepatitis B and hepatitis C virus infection induced liver cirrhosis [2]. Additionally, consumption of excessive alcohol or aflatoxin-contaminated food and all other cirrhosis-inducing conditions are also associated with HCC [3]. The molecular mechanisms driving hepatocarcinogenesis and progression have been extensively studied but are still not well defined. The integration of HBV DNA into the host genome results in local chromosomal aberrations, leading to the altered expression of cancer related genes [4]. It is apparent that specific growth factors may be involved in HCC progression. For example, IGF signaling is activated in HCC and IGF-1R blockage provides effective anti-tumor activity [5]. High levels of TGF-β and CXCR4 confer HCC cells with a mesenchymal-like phenotype, which contributes to tumor progression and dissemination [6]. Thus, growth factors orchestrate signaling pathways to regulate HCC progression.

Similar to other cancers, progression of HCC is considered to be governed by microenvironmental cues, including hypoxia [7, 8]. Hypoxia commonly develops within solid tumors due to dramatic cell proliferation and poor blood supply caused by inadequate or aberrant vasculature formation [8]. Furthermore, liver fibrosis caused by chronic injury subsequently results in structural and functional abnormalities of liver vasculature. The shortage of blood supply eventually leads to a hypoxia microenvironment in HCC [9]. It has been demonstrated that hypoxia enhances tumor progression through adaptive cellular programs promoting cell survival, motility, metabolism and tumor angiogenesis [8]. In addition, tumor hypoxia contributes to resistance to chemotherapy and radiotherapy by virtue of an enhanced CSC population [10, 11]. Moreover, CSCs promote relapse and metastasis of various tumors, including HCC [12, 13]. HCC patients with increased expression of EpCAM, a biomarker of HCC stem cells, exhibit increased metastasis and a significantly shorter survival [14]. Oncostatin M (OSM), an interleukin 6-related cytokine, is reported to promote hepatocyte differentiation of liver CSCs and increase the chemosensitivity of HCC [15]. It has also been shown that hypoxia regulates expression of VEGFA, IL-6 and CSC markers to maintain CSC functions in prostate cancer [16]. However, the precise mechanism by which hypoxia mediates the function of CSCs in HCC is not fully understood.

ARTN is one of four members of the glial-cell line-derived neurotrophic factor (GDNF) family of ligands (GFL). ARTN signaling is reported to be mediated through at least GFRα3, resulting in receptor tyrosine kinase RET signaling to downstream pathways [17]. Accumulating evidence indicates that ARTN possesses a critical role in cancer cell population adaptability to hostile challenges such as antiestrogens, Trastuzumab, chemotherapeutics and ionizing radiation [18–20]. The adaptive response mediated by ARTN to these therapeutic approaches involves an increase in the cancer stem cell population [19, 20].

In this study, we integrate *in vitro* and *in vivo* models to determine that hypoxia regulated ARTN promotes HCC progression. In so doing, we demonstrate that ARTN possesses a critical role in the hypoxia induced CSC expansion in HCC. Our study provides insights into the mechanism by which hypoxia promotes the CSC population.

## RESULTS

### High ARTN expression in HCC is associated with larger tumor size and poor survival outcome

**Figure 1:**
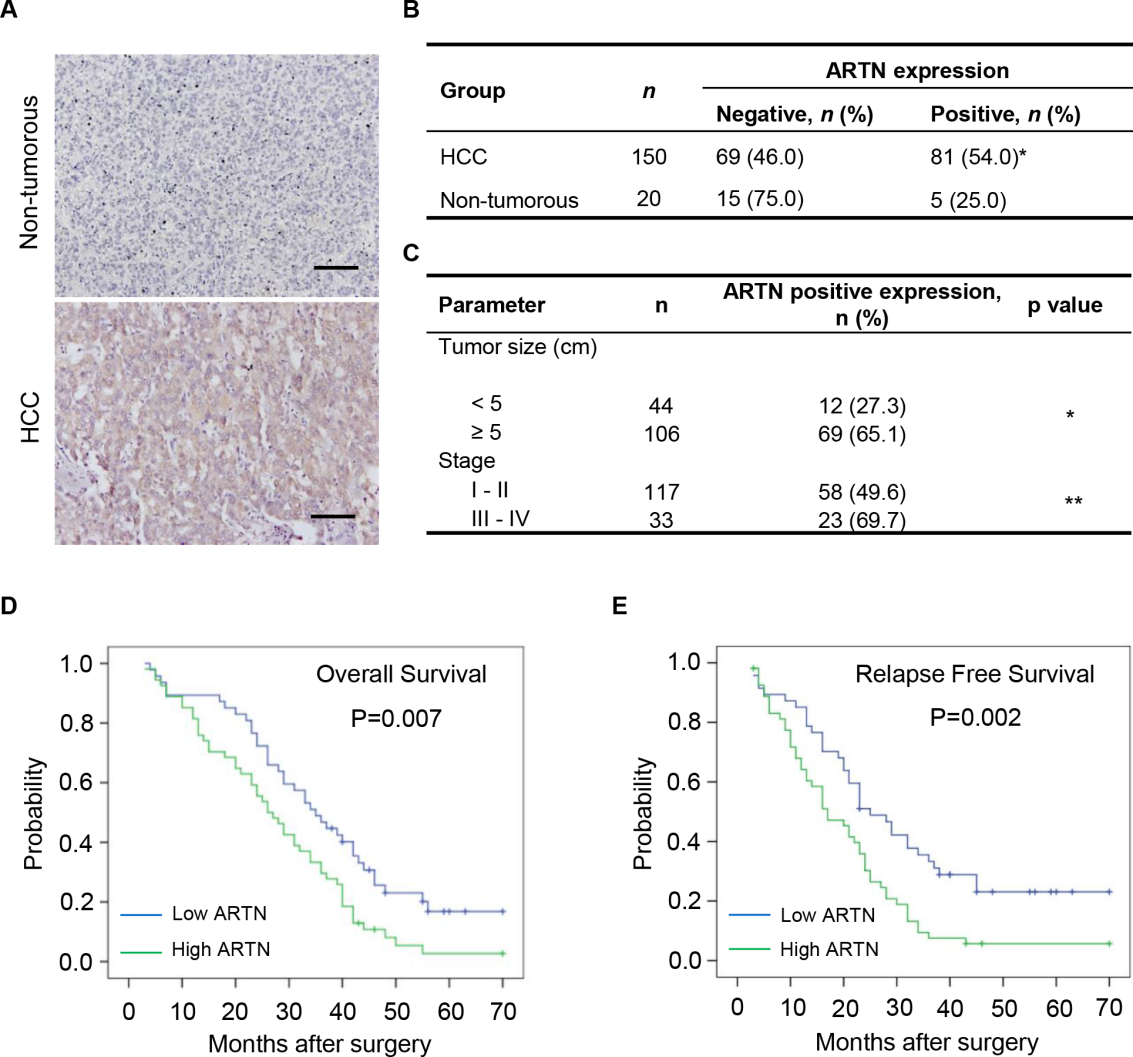
Increased ARTN expression is associated with poor prognosis (**A**–**B)** IHC analysis of ARTN expression levels in human primary HCC specimens and non-tumorous liver specimens. The representative pictures were shown at 200× magnification. **(C)** Correlation between ARTN expression and tumor size and histological grade of HCC. (**D**–**E)** The relationship of ARTN expression levels and overall survival (OS) or relapse free survival (RFS) of HCC patients by Kaplan-Meier analyses. Log rank test *p*-values were shown. *P < 0.05; **P < 0.01 (*χ*^2^ test).

In an attempt to define the clinical relevance of ARTN expression in HCC, we evaluated the abundance of ARTN protein in archived HCC specimens (*n* = 150) and adjacent non-tumorous liver tissues (*n* = 20) by immunohistochemistry (IHC). In HCC tissues that expressed ARTN, elevated ARTN protein was predominantly detected within the cytoplasm of HCC cells; (Figure 1A). The proportion of HCC specimens which exhibited positive ARTN IHC staining (54%) was more than two-fold that of adjacent non-tumorous liver specimens (25%, *P* < 0.05, Figure 1B). ARTN protein expression in hepatocellular carcinoma samples and the corresponding adjacent non-tumorous tissues was also specifically examined by IHC staining. Thirteen of twenty patients were positive for expression of ARTN protein in tumors compared with five of twenty adjacent non-tumorous tissues (*P* = 0.0284), which further exemplifies that the expression of ARTN is elevated in HCC (Supplementary Figure S1A). Furthermore, we determined whether ARTN expression was correlated with the clinicopathologic features and prognosis of HCC patients. High expression of ARTN was observed to be associated with larger tumor size (*P* < 0.05) and higher clinical stage in HCC patients (*P* < 0.01, Figure 1C). The lack of relationship between ARTN and other clinicopathological characteristics are summarized in Supplementary Figure S1B. Furthermore and shown in Supplementary Figure S1C, amongst all GDNF family members, only ARTN mRNA expression was significantly increased in HCC samples compared to normal liver tissues in a published HCC mRNA array dataset (GSE14323) [21]. To assess the relevance of ARTN to HCC patient survival, we performed Kaplan-Meier survival analyses in the HCC cohort. HCC patients with high expression of ARTN exhibited a shorter overall and relapse free survival compared with patients whose tumors expressed lower levels of ARTN protein (Figure 1D and 1E).

### ARTN enhances oncogenicity of HCC cells *in vitro* and *in vivo*

We first determined the mRNA expression levels of ARTN and its receptors in 7 HCC cell lines and the LO2 normal liver cell line. As observed in Supplementary Figure S1D, ARTN and its predominant receptors RET and GFRα3, were expressed in five cell lines (Bel7404, HepG2, Hep3B, LO2 and PLC). GFRα1, also a receptor for ARTN [17], was shown to be expressed in these cells except for LO2 cells (Supplementary Figure S1D). Based on this finding, Hep3B and HepG2 cell models with forced or depleted ARTN expression were established to assess the functional consequences of modulation of ARTN expression. Modulation of ARTN expression in these cells was determined at both mRNA and protein levels by RT-PCR and western blot, respectively (Supplementary Figure S1E and S1F).

**Figure 2:**
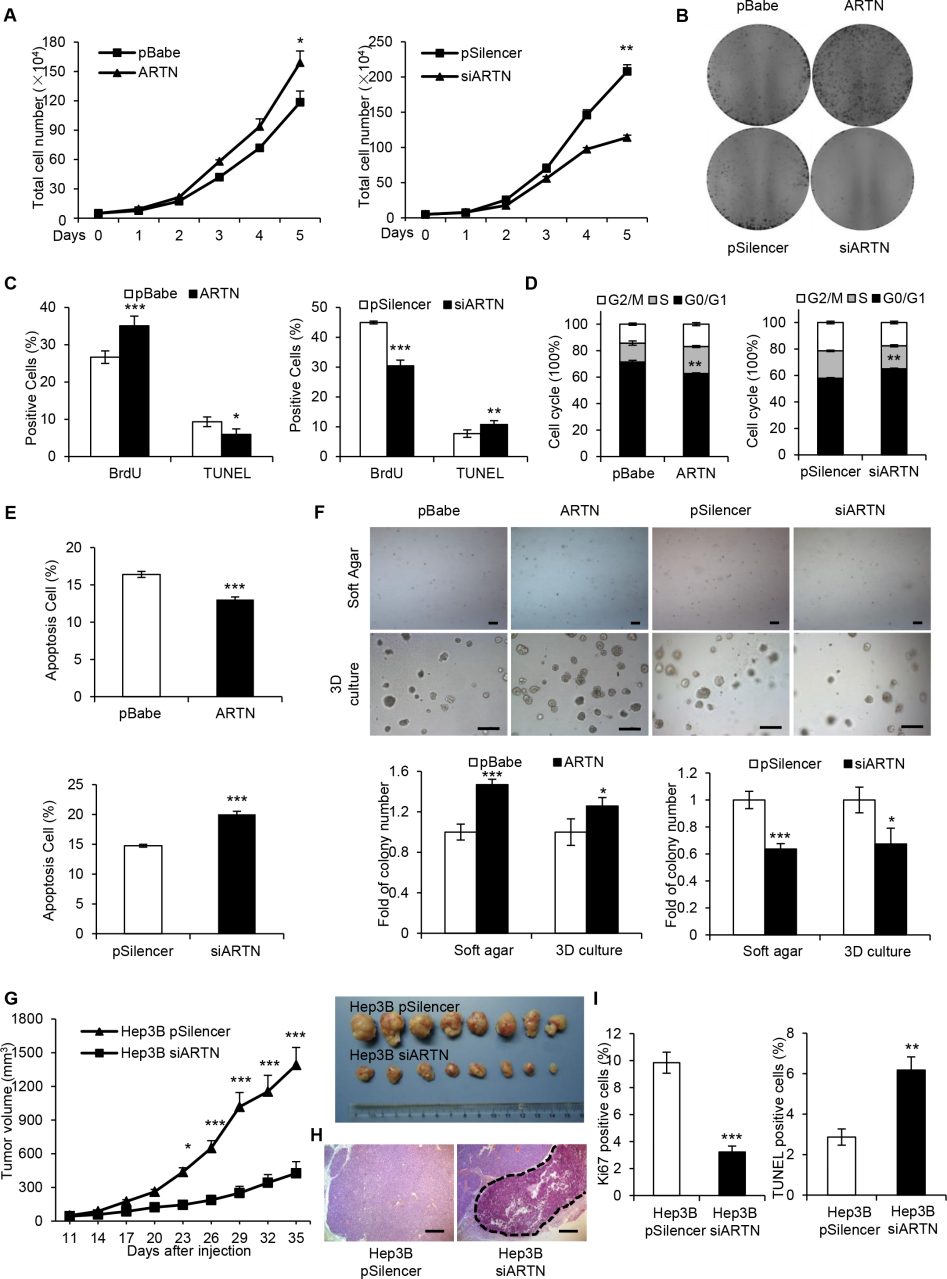
ARTN promotes oncogenicity of HCC cells (**A**) Proliferation of Hep3B cell lines was assessed by total cell number counting. (**B**) Foci formation of Hep3B cells in monolayer culture after two weeks. (**C**) The percentages of BrdU and TUNEL positive Hep3B cells. (**D**) Cell cycle analysis of Hep3B cells were assessed by flow cytometry after staining with PI. The percentages of cells in three phases are shown in histogram. (**E**) Apoptotic cells were detected by Annexin V and PI staining with flow cytometry. (**F**) Soft agar (Upper panels, 40×) and 3D Matrigel (Lower panels, 100×) colony formation. Colony numbers were counted and are shown in histogram. (**G**) Tumor growth curve of Hep3B-pSilencer and Hep3B-siARTN cells implanted into the flank of male nude mice. H, H & E staining of tumors formed by Hep3B-pSilencer and Hep3B-siARTN cells. The dotted line represented the necrotic areas. Magnification, 100×. (**I**) Cell proliferation and apoptosis were measured by Ki-67 staining and TUNEL assay on the sections of tumors. Mean ± SD, *n* = 3, *P < 0.05; **P < 0.01; ***P < 0.001.

We next examined the potential effects of ARTN on cell proliferation. Hep3B-ARTN cells exhibited increased total cell number compared with Hep3B-pBabe cells over a period of five days. Conversely, depletion of ARTN decreased cell number by approximately 25% compared with the control cells (Figure 2A). In foci formation assays, Hep3B-ARTN cells generated significantly more and larger colonies compared with control cells. In contrast, Hep3B-siARTN cells failed to form visible colonies after two weeks of culture despite formation of colonies by control cells (Figure 2B). BrdU incorporation and TUNEL assay were respectively employed to measure entry to S-phase and apoptosis in HCC cells. Hep3B cells with forced expression of ARTN exhibited a substantially increased population of cells with BrdU incorporation, suggestive of a higher rate of mitosis. In contrast, ARTN depletion impaired the ability of Hep3B cells to enter S-phase (Figure 2C). We also confirmed that ARTN increased the percentages of the G2 and S phase population in Hep3B cells by flow cytometry. In contrast, cell cycle progression was attenuated by depletion of ARTN (Figure 2D). Moreover, the percentage of TUNEL-positive and Annexin V-positive cells, representative of the apoptotic cell population were reduced in Hep3B-ARTN cells, whereas ARTN depletion increased the proportions of TUNEL and Annexin V positive cells (Figure 2C and 2E). Furthermore, ARTN increased the number and size of spheroids in soft agar and 3-dimensional (3D) Matrigel culture. Conversely, Hep3B-siARTN cells exhibited a decreased capacity to form spheroids in such conditions compared with Hep3B-pSilencer cells (Figure 2F). To exclude cell type specific effects, the same experimental approaches were adopted in HepG2 cells and concordant effects on cell behavior were observed (Supplementary Figure S2A–S2F). Thus, we conclude that ARTN enhances HCC cell proliferation, inhibits cell death and increases anchorage independent cell growth and growth in 3D matrigel.

To determine whether ARTN increased HCC growth *in vivo*, Hep3B-pSilencer and Hep3B-siARTN cells were injected into the flanks of BALB/c *nu/nu* male mice. At the end of 5 weeks, the tumors formed by ARTN depleted cells were strikingly smaller by at least three folds than the tumors from control cells (Figure 2G). Histologically, only tumors derived from Hep3B-siARTN cells showed massive necrosis determined by H & E staining whereas tumors derived from control cells did not (Figure 2H). Significantly reduced Ki-67 and elevated TUNEL labeling was observed in Hep3B-siARTN derived tumors indicative of decreased cell proliferation and increased apoptosis (Figure 2I). Additionally, Hep3B-pBabe and Hep3B-ARTN cells were subcutaneously implanted in male nude mice. After a period of 26 days, we observed that the tumors formed by Hep3B-ARTN cells were approximately 2-fold larger than those formed by Hep3B-pBabe cells. Moreover, the Hep3B-ARTN tumors exhibited higher percentages of Ki-67 positivity and a decreased proportion of TUNEL-positive cells compared with the Hep3B-pBabe tumors (Supplementary Figure S2G–S2I). Thus, modulation of ARTN expression influences HCC growth *in vivo*.

### ARTN increases the stem cell-like behavior and metastatic capacity of HCC cells

**Figure 3:**
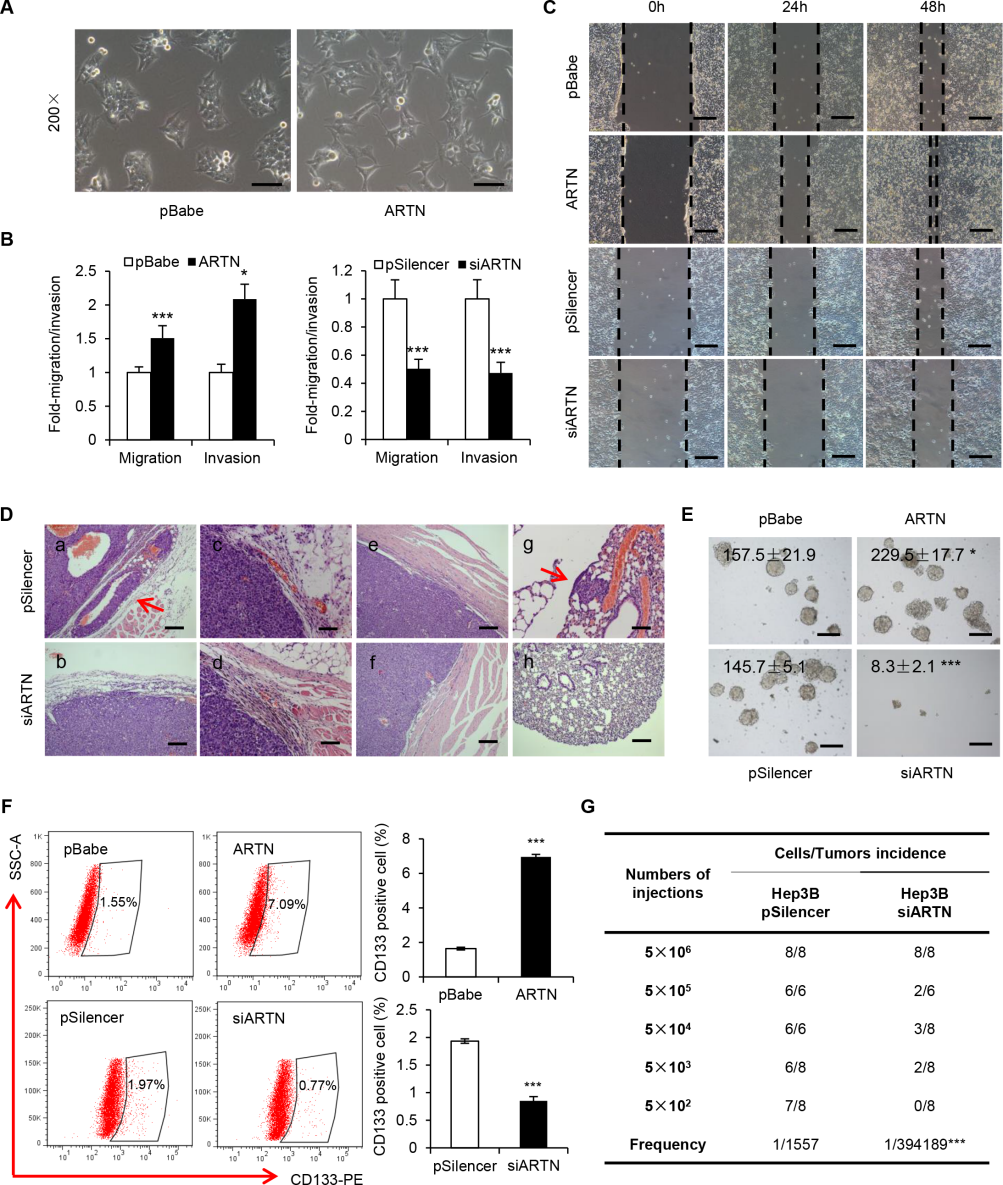
ARTN enhances the metastatic capacity and CSC properties of HCC cells (**A**) Morphology of Hep3B–ARTN cells and control cells. Representative pictures were captured using phase-contrast microscopy at 200 × magnification. (**B**) Transwell migration and invasion assay of Hep3B cells. (**C**) Wound healing assay of Hep3B-ARTN and Hep3B-siARTN cells compared with their respective control cells. Magnification, 100×. (**D**) H & E staining of primary tumors and lungs from mice xenograft model. Arrows indicated capsular invasion (a) and lung metastasis foci (g). (**E**) Tumorsphere formation of Hep3B cells. The total tumorsphere numbers in each well were counted and images were taken at 100× magnification. (**F**) CD133 positive cell population in Hep3B cells was determined by flow cytometry. Results are presented in histogram. (**G**) Tumor incidence of Hep3B cells stably transfected with siARTN construct and its control plasmid. Cells were injected into the flank of mice with limiting dilutions as indicated. The number of tumors formed in each group was counted after 4 weeks, and the CSC frequency was calculated using ELDA software. Mean ± SD, *n* = 3, *P < 0.05; ***P < 0.001 (Student *t* test for Figure 3B–3F, χ^2^ test for Figure 3G).

In monolayer culture, Hep3B-ARTN cells assumed a scattered and spindle-like morphology whereas control cells were tightly connected and exhibited an epithelial-like morphology, suggesting ARTN may regulate HCC cytoskeletal dynamics which is often linked to cell motility and metastatic potential (Figure 3A). Increased expression of ARTN in Hep3B cells promoted cell migration and invasion in transwell assays (Figure 3B). Additionally, in a wound healing assay, Hep3B-ARTN cells closed the wound much faster than Hep3B-pBabe cells (Figure 3C). In contrast, ARTN depletion resulted in a 2-fold reduction of cell migration and invasion; and slower wound closing compared to control cells (Figure 3B and 3C). This data was again repeated in HepG2 cells and consistent results were observed, suggesting that ARTN increases HCC cell motility *in vitro* (Supplementary Figure S3A–S3C).

We further examined whether ARTN would promote invasion and metastasis *in vivo*. Sections of the primary tumors and lungs from mice injected with Hep3B cells were examined by H&E staining. In contrast to Hep3B-pBabe cells, we observed obvious cellular invasion of venules and adjacent muscle tissue in tumors generated by Hep3B-ARTN cells (Supplementary Figure S3D). Furthermore, 6 of 7 mice injected with Hep3B-ARTN cells exhibited spontaneous pulmonary metastasis, whereas only one mouse of the control group showed metastatic lesions in the lungs (Supplementary Figure S3E). The number and size of micrometastasis were also increased in the lungs of mice bearing Hep3B-ARTN cell tumors (Supplementary Figure S3D and S3E). In addition, tumors from Hep3B-siARTN cells were well encapsulated and non-invasive, however, tumors of the other three groups displayed evidence of capsular invasion and occurrence of satellite nodules (Figure 3D and Supplementary Figure S3D). None of 8 mice bearing Hep3B-siARTN cell tumors exhibited spontaneous pulmonary metastasis in contrast to 2 of 8 in the control group (Supplementary Figure S3F). Hence, ARTN promoted HCC cell invasion to the surrounding tissues and metastasis to lungs *in vivo*.

The increase in anchorage independent growth by ARTN suggested that ARTN may promote self-renewal or tumor-initiating capacity (TIC). It is postulated that a small subset of cancer cells that exhibit stem cell-like properties are responsible to drive cancer progression and metastasis [13]. By extension, we therefore performed tumorsphere formation assays to determine if ARTN promoted a CSC-like phenotype in HCC cells. Hep3B-ARTN cells were observed to generate more tumorspheres than Hep3B-pBabe cells in conditioned tumorsphere culture medium in 6-well plates coated with Poly-HEMA (2-hydroxyethyl methacrylate). ARTN depletion dramatically decreased the number and size of Hep3B tumorspheres (Figure 3E). Given that the existence of CSCs is considered to be responsible for progression of HCC [12], several useful markers have been verified as effective predictors for the HCC CSC population. As demonstrated previously, CD133 has been identified to serve as a stem cell marker in HCC cells [22]. Forced expression of ARTN in Hep3B cells increased the CD133 positive population by more than 4 folds compared with control cells. In contrast, ARTN depletion in Hep3B cells reduced the CD133 positive population (Figure 3F). We also repeated the same experiments mentioned above in HepG2 cells and consistent results were observed, indicating that ARTN promotes the stem cell-like behavior of HCC cells *in vitro* (Supplementary Figure S3G and S3H). To determine the role of ARTN on TIC *in vivo*, Hep3B-pSilencer/siARTN cells were subcutaneously injected into the mice in a dilution series from 5×10^5^ to 500 cells. As summarized in Figure 3G, no tumors were observed in mice injected with 500 Hep3B-siARTN cells, whereas Hep3B-pSilencer cells showed a much higher tumor incidence (7 of 8 mice) at the same cell numbers. We also calculated the frequencies of CSCs using the extreme limiting dilution software [23]. ARTN knockdown dramatically decreased the CSC frequency (1/394189) compared with control cells (1/1557, *P* < 0.001), consistent with a role of ARTN in TIC in HCC cells.

Given that Hep3B and HepG2 cells are all cancer cell lines, we further explored the functional role of ARTN in immortalized and non-transformed human liver cells. *In vitro* gain-of-function and loss-of-function analyses were exploited in LO2 cells stably transfected with the pBabe-ARTN or pSilencer-siARTN constructs as compared with their control cells. The stable cell lines were verified by assessing the level of ARTN mRNA by RT-PCR (Supplementary Figure S4A). LO2-ARTN cells exhibited almost no alternation in cell viability compared with LO2-pBabe cells, whereas ARTN depletion in LO2 cells negligibly decreased cell viability even after five days culture (Supplementary Figure S4B). Importantly, neither forced expression of ARTN nor ARTN depletion in LO2 cells affected colony generation in soft agar (Supplementary Figure S4C). Furthermore, there were no significant differences between LO2-ARTN or LO2-siARTN and their respective control cells in tumorsphere formation (Supplementary Figure S4D). Hence, ARTN apparently exerts differential functional properties in normal liver cells compared to cancer cells.

### ARTN promotes HCC progression via regulation of EMT and CSC pathway

**Figure 4:**
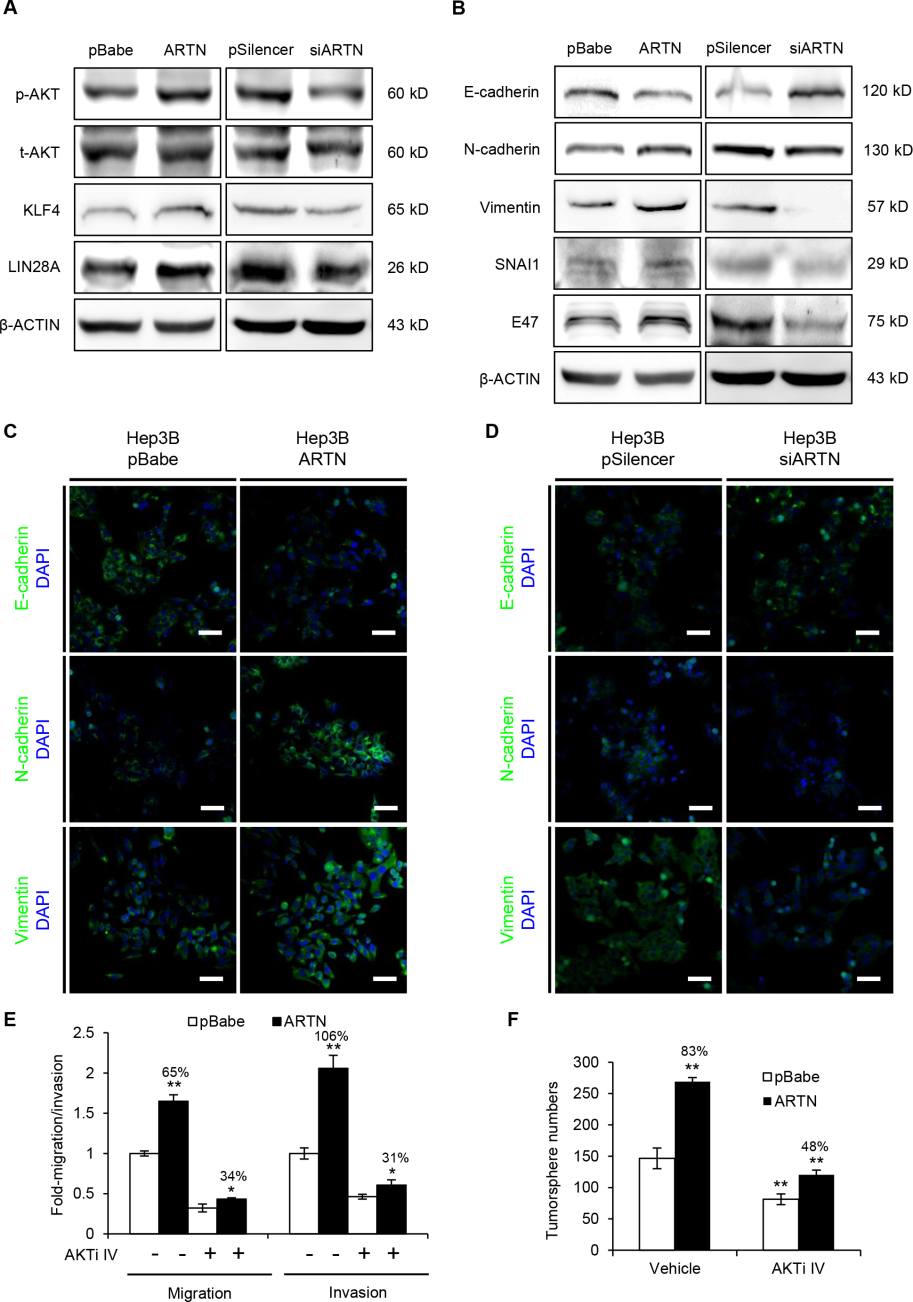
ARTN modulates multiple critical factors of CSC and EMT signaling (**A**) Western blot analysis for p-AKT and transcription factors (KLF4, LIN28A) in the CSC pathway. (**B**) Western blot analysis of EMT markers and inducers in Hep3B cells. (**C–D**) Immunofluorescence staining of E-cadherin, N-cadherin and Vimentin in Hep3B cells. Representative pictures were taken using confocal microscopy at 200 × magnification. (**E**) Migration and invasion assays in Hep3B-pBabe and Hep3B-ARTN cells treated with the specific AKT inhibitor IV (AKTi IV) or DMSO. (**F**) Tumorsphere formation of Hep3B-pBabe and Hep3B-ARTN cells treated with the specific AKT inhibitor IV or DMSO. Mean ± SD, *n* = 3, *P < 0.05; **P < 0.01 (ANOVA test).

Our data demonstrated that ARTN markedly promoted oncogenicity, metastasis and stem cell-like behavior in HCC cells. We therefore next explored whether ARTN regulated the signaling pathways involved in CSC and EMT, which are considered to be critical steps for cell motility and cancer metastasis [24]. It has been widely accepted that the activation of the AKT pathway enhances the CSC population and AKT is reported to be activated by RET, a receptor tyrosine kinase transducing the function of ARTN [17, 25]. In line with these reports, elevated p-AKT levels were detected in Hep3B–ARTN cells compared to the control cells. KLF4 and LIN28A, which are known as important cancer stem cell factors [26], were also increased in expression (Figure 4A, left panel). Conversely, AKT activation was diminished in Hep3B-siARTN cells, and similar to the observed decrease in KLF4 and LIN28A expression (Figure 4A, right panel). We also investigated the effect of ARTN on expression of some important proteins involved in the EMT process. Loss of epithelial marker (E-cadherin) and increase of mesenchymal markers (Vimentin and N-cadherin) are the characteristic of EMT [24, 27]. SNAI1 and E47 are considered as EMT inducers as they are strong repressors of E-cadherin [27]. Indeed, we observed a decrease of E-cadherin protein expression in cells with forced expression of ARTN, whereas E-cadherin was increased in ARTN depleted cells. Vimentin and N-cadherin were increased consequent to forced expression of ARTN and decreased upon depletion of ARTN as were SNAIl and E47 (Figure 4B). The expression of EMT markers was also assessed by immunofluorescence. Consistent with the immunoblot results, forced expression or depletion of ARTN also respectively increased or inhibited EMT markers as observed by immunofluorescence, suggesting that ARTN orchestrates EMT (Figure 4C and 4D). Similar results were also obtained in HepG2 cells (Supplementary Figure S5A–S5D).

AKT activation has been demonstrated to increase SNAI1 expression, which promotes EMT and the CSC-like phenotype in cancer [28]. Thus, AKT signaling might be required for EMT and CSC maintenance by ARTN. To this end, Hep3B-pBabe and Hep3B-ARTN cells were both treated with 100 nM AKT inhibitor IV, a specific inhibitor of AKT which clearly diminished the phosphorylation of AKT (Supplementary Figure S5E). In transwell assays, AKT inhibition significantly abolished the promotion of Hep3B cell migration (from 65% to 34%) and invasiveness (from 106% to 31%) by ARTN (Figure 4E). Furthermore, the enhancement of tumorsphere formation in Hep3B-ARTN cells was greatly impaired when p-AKT activity was blocked (from 83% to 48%, Figure 4F). Hence, ARTN promotes the metastatic properties and tumor-initiating capacity of HCC cells by AKT modulation of factors involved in EMT and stemness.

### Hypoxia induced factor-1α (HIF-1α) directly controls ARTN expression

**Figure 5:**
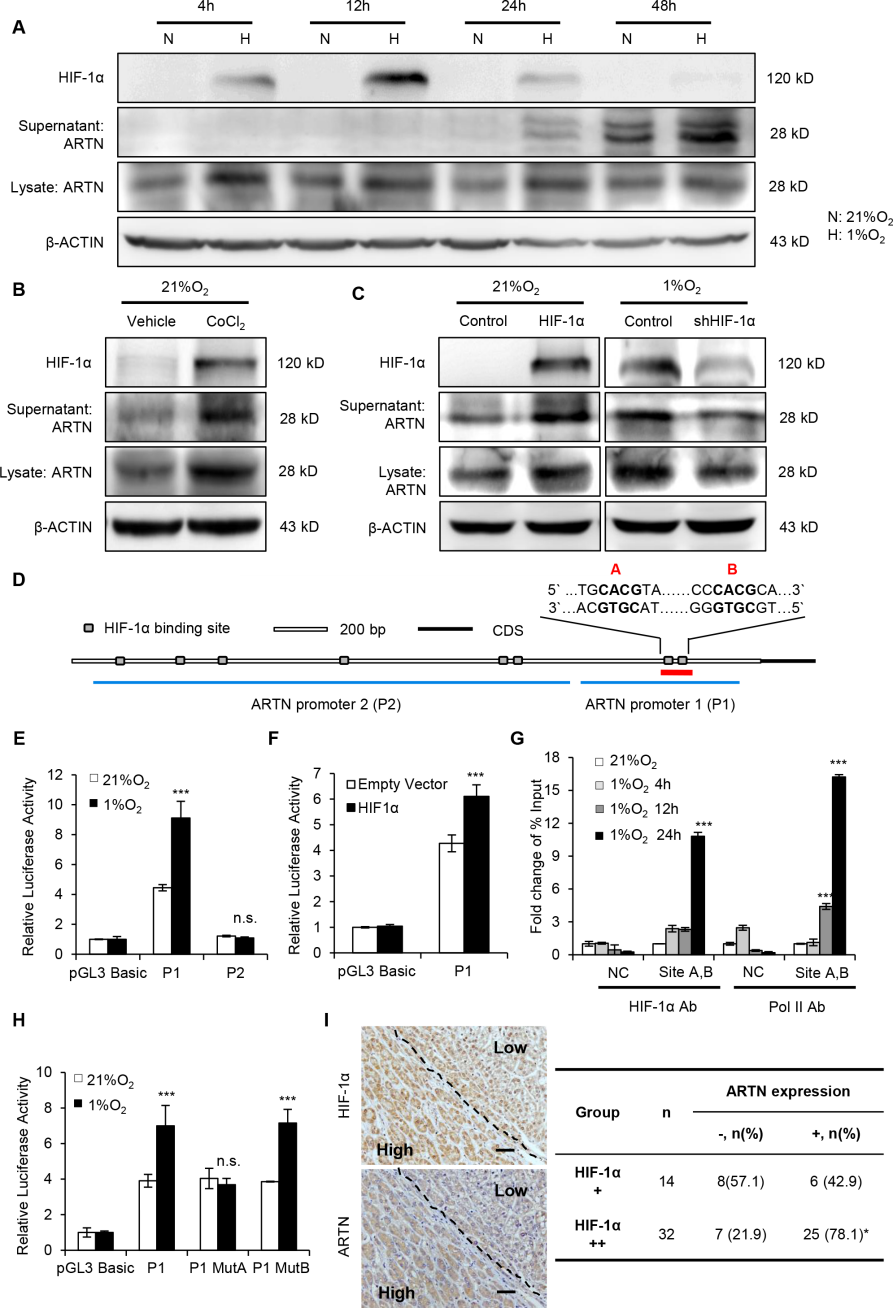
Hypoxia transcriptionally enhances ARTN expression (**A**) Time-course analysis of ARTN and HIF-1α protein under normoxia and hypoxia. Hep3B cells were incubated in normoxic or hypoxic condition for every indicated time. ARTN and HIF-1α expression was determined by Western blot. N, 21% O_2_; H, 1% O_2_. (**B**) Western blot analysis. Hep3B cells were treated with CoCl_2_ for 48 hours under normoxic culture. (**C**) The relationship of ARTN expression and HIF-1α, determined by western blot. In normoxia, Hep3B cells were transiently transfected with HIF-1α cDNA or the control construct; or HIF-1α was depleted by shRNA plasmid under hypoxia for 48 hours. (**D**) Bioinformatic analysis of predicted binding sites for HIF-1α (grey box) in the ∼3-kb regulatory region upstream of the ARTN start codon. (**E**) Luciferase activity of ARTN promoter. Hep3B cells were transfected with the various promoter constructs (P1, P2) and then incubated in normoxic (21% O_2_) or hypoxic (1% O_2_) conditions. (**F**) Analysis of promoter (P1) activity in Hep3B cells after transfection of HIF-1α cDNA construct and its control under normoxia. (**G**) ChIP assays of Hep3B cells with 0-24 hours hypoxic treatment. qPCR was carried out with specific primers for the last two sites (sites A and B). The histogram shows the fold change of relative enrichment to the input. (**H**) Analysis of promoter (P1) activity in Hep3B cells transfected with ARTN promoter construct (P1) or the two mutant promoter constructs (MutA, MutB) under normoxic (21% O_2_) or hypoxic (1% O_2_) conditions. (**I**) The correlation between ARTN and HIF-1α was assessed by IHC in HCC specimens. Represent pictures showed the co-expression of HIF-1α and ARTN in HCC specimens. Magnification, 100×. Mean ± SD, *n* = 3, *P < 0.05; ***P < 0.001; n.s. not significant (ANOVA test for Figure 5E–5H, χ^2^ test for Figure 5I).

Accumulating evidence suggests that a hypoxic microenvironment promotes CSC properties in several cancers, including HCC [10, 29]. To determine the possible response of ARTN to hypoxia in HCC, Hep3B cells were cultured in normoxic (21% O_2_) or hypoxic (1% O_2_) conditions for different times. Total mRNAs were collected and the expression of ARTN mRNA was analyzed by RT-PCR. As observed (Supplementary Figure S6A), the mRNA level of ARTN progressively increased under hypoxic conditions with the highest expression observed after 24 hours of hypoxic culture. The expression pattern of ARTN mRNA under hypoxic conditions was similar to VEGFA, a known hypoxia-inducible gene [30]. Next, we examined the protein level of ARTN under hypoxia. To exclude the effects of different cell confluence on total protein levels, both supernatant and cell lysate of Hep3B cells cultured in normoxia or hypoxia were collected at each time point. Immunoblots revealed both secreted and cellular ARTN protein were increased after 24 hours exposure to hypoxia (Figure 5A). Furthermore, treatment of cells with CoCl_2_, which mimics hypoxia *in vitro* [31], also increased ARTN protein expression (Figure 5B). Similar induction of ARTN mRNA and protein by hypoxia were also observed in HepG2 cells (Supplementary Figure S6B and S6C).

Transcriptional responses to hypoxia in tumors are predominantly mediated by the hypoxia-inducible factors (HIFs), especially HIF-1α [32]. To delineate how hypoxia regulates ARTN expression, we forced the expression of HIF-1α in Hep3B cells under normoxic conditions. Forced expression of HIF-1α under normoxia also increased the ARTN protein (Figure 5C). Conversely, depletion of HIF-1α expression in hypoxia, using a specific shRNA construct, decreased the protein level of ARTN both in cell lysate and supernatant (Figure 5C). Similar results were observed in HepG2 cells (Supplementary Figure S6D). Hence, increased expression of ARTN under hypoxia is mediated by HIF-1α.

rVista 2.0 predicted potential binding sites of HIF- 1α in the 3kb region upstream of the ARTN start codon [33]. This region contains eight putative hypoxia-response elements (HREs) which consist of the core HIF-1α binding site 5′-CGTG-3′ and lacks the binding sites of HIF-2, another important transcriptional factor induced in hypoxia [34]. Two DNA fragments spanning these HREs were inserted into the pGL3 reporter plasmid, namely ARTN promoter 1 (P1, 900bps), containing the two highest-scored HREs (site A and site B) and ARTN promoter 2 (P2, 1900bps) containing the other six HREs, respectively (Figure 5D). Hep3B cells were transfected with these two plasmids and then cultured in normoxic or hypoxic conditions for 48 hours. Insertion of the P1 sequence into the pGL3 reporter plasmid enhanced luciferase activity in normoxia by four folds (Figure 5E). Furthermore, the luciferase activity of the P1 plasmid was increased two folds in hypoxic conditions compared to that of normoxia. However, there was no evident alternation of luciferase activity in P2 plasmid transfection, either in normoxia or in hypoxia (Figure 5E). Thus, we focused on how hypoxia regulated P1 transcriptional activity. Consistent with HIF-1α upregulation of ARTN in normoxia, forced expression of HIF-1α in normoxia increased the luciferase activity of the P1 promoter (Figure 5F). Similar enhancement of P1 luciferase activity was observed in hypoxia treated or HIF-1α overexpressed HepG2 cells (Supplementary Figure S6E and S6F). Next, ChIP assays were performed to determine whether HIF-1α bound to the putative sites of promoter P1. Hep3B cells were cultured in normoxia for 24 hours (0 h) or hypoxic condition for 4, 12 and 24 hours and genomic DNA was collected by using anti-HIF-1α or anti-PolII antibodies. Analysis of DNA enrichment by qPCR revealed a significant enrichment of HIF-1α around the site A/B DNA regions after incubation in hypoxia for 24 hours. Moreover, an increased RNA polymerase II (PolII) occupation was also observed around the site A/B, indicating the recruitment of the transcription complex at the ARTN promoter under hypoxia (Figure 5G). A chromosome 12p13.3 region, which is considered to possess no transcription factor binding sites, was used as negative control in ChIP assay [35]. We next deleted the two binding sites of promoter P1 respectively and tested the luciferase activity of each mutant P1 plasmid. As shown, only mutation of site A abrogated the luciferase activity of the P1 response to hypoxia (Figure 5H). Next, we determined if a correlation between HIF-1α and ARTN protein expression existed in HCC patients. IHC staining of HIF-1α or ARTN were performed on parallel slices. Figure 5I showed representative fields in which co-localization of HIF-1α and ARTN was observed. As shown, the portion with higher HIF-1α expression in HCC also corresponded to high ARTN expression. Moreover, we observed a positive correlation between HIF-1α and ARTN expression with statistical significance (Figure 5I, *P* < 0.05, Pearson *r* = 0.3461).

### Hypoxia induced AKT activation and CSC expansion in HCC is ARTN dependent

**Figure 6:**
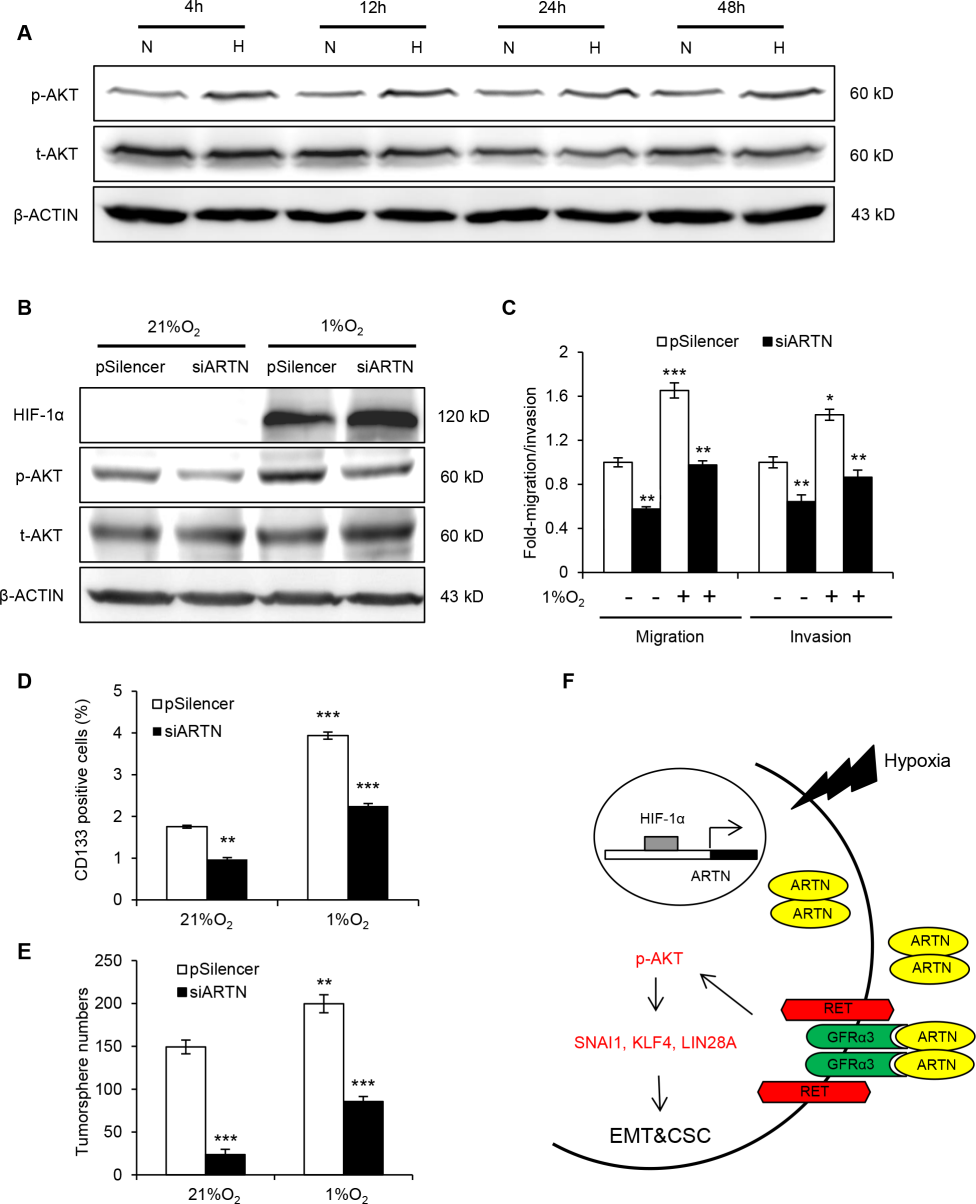
ARTN mediates hypoxia induced EMT and CSC promotion in HCC cells (**A**) Western blot analysis of p-AKT level under hypoxia treatment. (**B**) Western blot analysis of p-AKT level. Hep3B-pSilencer and siARTN cells were both subjected to normoxic (21% O_2_) and hypoxic (1% O_2_) conditions. (**C**) Migration and invasion assays in Hep3B-pSilencer and Hep3B-siARTN cells under normoxic or hypoxic conditions. (**D**) CD133 positive population in Hep3B-pSilencer and Hep3B-siARTN cells under normoxic or hypoxic condition was determined by flow cytometry. (**E**) Quantification of tumorsphere formation in Hep3B-pSilencer and Hep3B-siARTN cells treated under normoxic or hypoxic condition. (**F**) A schematic illustration of the proposed model of ARTN regulating EMT and CSC properties of HCC cells under hypoxia. Mean ± SD, *n* = 3, *P < 0.05; **P < 0.01; ***P < 0.001 (ANOVA test).

Activated AKT modulates numerous cellular processes in cancer, including proliferation, metastasis and drug resistance [36]. Furthermore, the activation of AKT signaling has been shown to promote hypoxia induced breast CSCs expansion [29]. As predicted, hypoxia treatment resulted in an increased p-AKT at each time point compared with corresponding normoxia (Figure 6A and Supplementary Figure S6C).

The effects of ARTN on AKT activation via RET in normal culture condition suggested ARTN might be a key regulator of hypoxia promoted AKT activation. p-AKT was significantly diminished by ARTN depletion in hypoxia, remaining at a similar level to control cells in normoxia (Figure 6B). A series of experiments were performed to identify the functional role of the hypoxia/HIF-1α/ARTN axis in regulating HCC cell motility and CSC properties. Hypoxia treatment significantly enhanced the migration and invasion of Hep3B cells, whereas ARTN depletion impaired Hep3B cell migration and invasion both in 21% O_2_ and 1% O_2_ conditions (Figure 6C). Hypoxia promoted the CD133 positive population in Hep3B cells; and ARTN depletion decreased CD133 positive cells both in normoxic and hypoxic condition (Figure 6D). Cells incubated under hypoxia formed more tumorspheres than those under normoxia. Depletion of ARTN diminished tumorsphere formation in Hep3B cells under both normoxic and hypoxic culture (Figure 6E). In summary, our study has described a novel HIF-1α/ARTN/AKT axis regulating the metastasis and CSC capacity in HCC as shown in the model in Figure 6F.

## DISCUSSION

Sustained proliferative signaling and survival are hallmarks of cancer. Growth factor ligands produced by cancer cells or tumor-associated stroma, activate downstream signaling pathways via their cognate receptors, resulting in autocrine or paracrine stimulation proliferation and survival [37]. There is growing evidence of an association between aberrant secretion of growth factors and exacerbated progression of HCC [5, 6]. However, the functions of members of the GDNF family of ligands, including ARTN, and their receptors in HCC are still largely unknown. We demonstrated herein that increased ARTN expression was associated with a higher clinical stage and worse outcome in HCC patients. Interestingly, ARTN was the only member of the GFL family observed to be increased in expression in HCC. Furthermore, the mRNA levels of RET, and GFRα1–4 were also not increased in a published HCC mRNA array dataset (GSE14323). There is however some evidence that components of the GFL signaling pathways are involved in HCC. For example, 3 SNPs in GFRα1, one of the receptors for ARTN were indentified to be associated with HCC susceptibility [38]. A recent study has also reported that RET, the receptor tyrosine kinase utilized by GFL ligands, is activated in some HCC cell lines [39]. Furthermore, Sorafenib (BAY 43–9006), a multi-kinase inhibitor also targeting RET, has potent anti-tumor activity in HCC clinical trials [40]. However, a recent report has demonstrated that ARTN also interacts with syndecan-3(SDC3) and triggers downstream pathways via src kinase activation, indicating that ARTN also functions in a RET-independent manner [41]. It has also been demonstrated that ARTN promotion of the CSC population in ER- mammary carcinoma cells is equally efficacious in RET positive or RET negative cells [20]. Given that ARTN stimulated cellular function is also RET-independent, the preferred approach to inhibit ARTN function in HCC would be by targeting the ligand itself, for example, as achieved with the neutralizing monoclonal antibody Bevacizumab for VEGFA, which also binds multiple receptors [42]. Moreover, we observed that ARTN depletion did not alter the functional properties of normal liver cells indicative that there may be minimal toxicity from therapeutic inhibition of ARTN in HCC and mouse models of ARTN deficiency support this speculation [43]. However, the mechanisms as to why ARTN only modulated HCC cell function, and whether this notion would also be observed *in vivo*, warrants further study. It is noteworthy that high ARTN mRNA expression was also observed in immortalized normal liver cells LO2. One explanation may be that the LO2 cell line is derived from embryonic liver tissue [44] and the gene expression profile of embryonic cells is similar to that of cancer cells compared with differentiated cells and especially the genes associated with EMT and cell motility [45].

Recent studies have demonstrated a pivotal functional role of ARTN in acquired resistance to multiple therapeutic approaches utilized in mammary carcinoma. For example, ARTN is an estrogen-regulated gene and mediates acquired tamoxifen resistance by increasing BCL-2 expression in ER-positive breast cancer [18]. It has been also reported that ARTN confers chemo- and radio-resistance upon mammary carcinoma cells by promoting TWIST1-BCL-2-dependent CSC-like behavior [20]. Furthermore, depletion of ARTN restores Trastuzumab sensitivity in Trastuzumab resistant HER2-positive mammary carcinoma cells [19]. Analogously, GDNF has recently been reported to function in a paracrine manner to promote a prostate tumor cell resistant phenotype in response to ionizing radiation and cytotoxic agents [46]. Based on our findings, ARTN signaling also possesses a critical role in HCC progression. ARTN activation of AKT was previously demonstrated to promote the expression of BCL-2 and TWIST1 resulting in enhanced oncogenicity and an increased CSC population in breast cancer [20, 47]. This signaling may also exist in the HCC model. Hypoxia induces a number of genes required for promotion of cancer progression [11].

To our knowledge, this work is the first demonstration that ARTN is a hypoxia-regulated gene in HCC. The ARTN receptor GFRα1 has also been reported to be regulated by hypoxia at the mRNA level [48], suggesting enhanced ARTN signaling in the hypoxic microenvironment. It has been reported that hypoxia drives breast CSC behavior and limits the effectiveness of antiangiogenic agents such as Sunitinib which is a VEGFA receptor tyrosine kinase inhibitor [29]. Moreover, our study indicated hypoxia-regulated ARTN promotes CSC functions in HCC. These observations lend credence to the speculation that ARTN may be a generalized response of tumor cells to a hostile microenvironment. A recent report indicated hypoxia induced CSC expansion is mediated through AKT/β-catenin signaling in breast cancer, which is consistent with our conclusion that hypoxia responsive ARTN promoted HCC CSC properties via activation of AKT [29]. It is proposed that hypoxic conditions promotes CSC properties in renal carcinoma by stimulating the expression of pluripotent stem cell gene OCT4 [11]. We have demonstrated herein that ARTN increases the protein levels of important CSC markers such as KLF4 and LIN28A which are also hypoxia regulated genes [11, 49]. Recently it was indicated that TGF-β, a distant relative of GFLs, is induced by hypoxia and associated with self renewal of CSC in HCC [9, 17, 50]. In addition, VEGFA, a well-known hypoxia-inducible growth factor, is reported to both promote angiogenesis and maintain CSC renewal in squamous skin tumors [30, 51]. It is of note that we have previously demonstrated that ARTN enhances VEGFA expression with consequent promotion of *de novo* angiogenesis [52] and increased VEGFA expression in hypoxia may therefore be partially mediated by ARTN. Hence, ARTN is hypoxia responsive and promotes an enhanced CSC population in hypoxic microenvironments in HCC.

## MATERIALS AND METHODS

### Plasmid constructs

The coding sequence of human ARTN (GenBank accession number NM_057090) was cloned into pBabe-puro plasmid. ARTN specific siRNA plasmid pSilencer-siARTN was constructed as described previously [53]. The HIF-1α overexpression construct was a kind gift from Huafeng Zhang. The shHIF-1α plasmid used in this study was obtained from The RNAi Consortium (TRC, MISSION^®^ TRC shRNA library, Sigma). For the ARTN, two DNA fragments containing HIF-1α binding sites upstream of ARTN initial codon were cloned into pGL3-Basic vector (Promega). All the mutant promoter constructs were generated using the QuickChange site-directed mutagenesis kit (Stratagene) and confirmed by DNA sequencing. All the primers for plasmid constructions were listed in Supplementary Table S1.

### Cell culture and reagents

Human HCC cell lines HepG2 and Hep3B were obtained from ATCC (Rockville, MD, USA). Bel-7404, LO2, PLC, QGY-7701, QGY-7703 and SMMC-7721 cells were kind gifts from Dr. Lijian Hui (Institute of Biochemistry and Cell Biology, Chinese Academy of Sciences). All cells were cultured in DMEM (Hyclone, Beijing, China) supplemented with 10% fetal bovine serum (Hyclone, Beijing, China), 1% penicillin/streptomycin (Gibco, Auckland, New Zealand). For tumorsphere assay, cells were cultured in DMEM/F12 (Invitrogen) supplemented with B27 (1:50; Gibco), BSA (0.4%; Sigma), EGF (20ng/ml; Upstate), bFGF (20ng/ml; Peprotech), insulin (5ug/ml; Sigma), penicillin-streptomycin (Gibco). HepG2, Hep3B and LO2 cells were stably transfected with pBabe puro-ARTN or the empty vector to generate ARTN and control cell lines, respectively. Similarly, the ARTN depleted cell lines and their control cell lines were produced by stably transfection with pSilencer-siARTN and pSilencer-CK, respectively. AKT inhibitor IV was from Calbiochem. Propidium iodide (PI) and CoCl_2_ was purchased from Sigma-Aldrich.

### RNA isolation and RT-PCR

Total RNA was isolated using Trizol (Invitrogen, Carlsbad, CA) according to the manufacturer's introduction. Reverse transcription PCR was performed by using RevertAid M-MuLV Reverse Transcriptase (Fermentas, St Leon-Rot, Germany). PCR was performed as described earlier [54]. Primers used in RT-PCR were listed in Supplementary Table S1.

### Cell function assays and flow cytometer analysis

Total cell number counting, BrdU incorporation, cell cycle analysis, TUNEL, Annexin V, foci formation, soft agar colony formation assay, 3D-matrigel culture, *in vitro* cell motility (migration, invasion and wound healing) assays and tumorsphere formation assay were carried out as previously described [55–57]. All the images were taken using a phase microscope (Olympus, Tokyo, Japan). The CD133 positive population in HCC cells was examined by using PE-conjugated monoclonal mouse anti-human CD133/1 (AC133, Miltenyi Biotec; Auburn CA).

### Western Blot analysis and immunofluorescence

Western blot analysis on cell lysate and supernatant was carried out as previously described and read on ImageQuant LAS4000 (GE Healthy) [55]. Immunofluorescence staining assay was performed as previously described. All antibodies used in western blot and immunofluorescence are listed in Supplementary Table S2.

### Reporter assay

Cells were seeded at about 60% confluence in 24-well plates. For the promoter analysis, 0.2 μg pGL3 Basic luciferase reporters and HIF-1α plasmid were co-transfected with an internal control plasmid pRL-TK into cells using Lipofectamine 2000 (Invitrogen). Cells were cultured in 21% O_2_ conditions or exposed to 1% O_2_ for 48 hours and then harvested to detect the Firefly and Renilla luciferase activities using the Dual-Luciferase^®^ Reporter Assay System (Promega).

### ChIP assay

Cells were harvested after culture in 21% O_2_ or 1% O_2_ for 4, 12 and 24 hours in 10 cm dishes. ChIP assay was carried out by using EZ ChIP kit (Upstate, Lake Placid, NY) following the manufacturer's instructions. The primers and antibodies used in ChIP assays were listed in the Supplementary Table S1 and S2.

### Histopathology

A total of 150 patients with primary HCC were enrolled in this study with informed patient consent and followed the human study protocol approved by Anhui Medical University Ethics Committee. Formalin-fixed and paraffin-embedded HCC and normal liver specimens were sourced from the Department of Pathology within the First Affiliated Hospital of Anhui Medical University (Hefei, Anhui, People's Republic of China) between 2004 and 2007. The histological type was assigned according to the criteria of the WHO classification system [58]. IHC was carried out as described [55, 59]. All the antibodies used in IHC were listed in Supplementary Table S2.

### Tumor xenograft in nude mice

Hep3B-pBabe/ARTN and Hep3B-pSilencer/siARTN cells were subcutaneously injected into the flank of 5-week-old BALB/c nude male mice (Shanghai Slaccas Co, Shanghai, China). For limiting dilution assay, a series of different number of control cells and siARTN cells were injected into host male mice. CSC frequency was calculated as described previously [23]. Tumor volume detection, histological analysis with H & E staining, Ki-67 and TUNEL immunostaining were performed as described earlier [54].

### Statistics analysis

All experiments were repeated at least three times, and all data are expressed as mean ± SD. If not specified otherwise GraphPad Prism (San Diego, CA) was used for statistical analysis of data. The tumor growth curves were analyzed using two-way ANOVA test with a Bonferroni posttest. The chi-squared (χ2) test was performed using SPSS (Chicago, IL). In all cases, *P* < 0.05 was considered significant.

## SUPPLEMENTARY MATERIALS FIGURES AND TABLES



## References

[1] Mittal S, El-Serag HB (2013). Epidemiology of hepatocellular carcinoma: consider the population. Journal of clinical gastroenterology.

[2] Branda M, Wands JR (2006). Signal transduction cascades and hepatitis B and C related hepatocellular carcinoma. Hepatology.

[3] Farazi PA, DePinho RA (2006). Hepatocellular carcinoma pathogenesis: from genes to environment. Nature reviews Cancer.

[4] Brechot C (2004). Pathogenesis of hepatitis B virus-related hepatocellular carcinoma: Old and new paradigms. Gastroenterology.

[5] Tovar V, Alsinet C, Villanueva A, Hoshida Y, Chiang DY, Sole M, Thung S, Moyano S, Toffanin S, Minguez B, Cabellos L, Peix J, Schwartz M (2010). IGF activation in a molecular subclass of hepatocellular carcinoma and pre-clinical efficacy of IGF-1R blockage. Journal of Hepatology.

[6] Bertran E, Crosas-Molist E, Sancho P, Caja L, Lopez-Luque J, Navarro E, Egea G, Lastra R, Serrano T, Ramos E, Fabregat I (2013). Overactivation of the TGF-beta pathway confers a mesenchymal-like phenotype and CXCR4-dependent migratory properties to liver tumor cells. Hepatology.

[7] Dai CX, Gao Q, Qiu SJ, Ju MJ, Cai MY, Xu YF, Zhou J, Zhang BH, Fan J (2009). Hypoxia-inducible factor-1 alpha, in association with inflammation, angiogenesis and MYC, is a critical prognostic factor in patients with HCC after surgery. BMC cancer.

[8] Pouyssegur J, Dayan F, Mazure NM (2006). Hypoxia signalling in cancer and approaches to enforce tumour regression. Nature.

[9] Kim KR, Moon HE, Kim KW (2002). Hypoxia-induced angiogenesis in human hepatocellular carcinoma. Journal of molecular medicine.

[10] Heddleston JM, Li ZZ, McLendon RE, Hjelmeland AB, Rich JN (2009). The hypoxic microenvironment maintains glioblastoma stem cells and promotes reprogramming towards a cancer stem cell phenotype. Cell cycle.

[11] Keith B, Simon MC (2007). Hypoxia-inducible factors, stem cells, and cancer. Cell.

[12] Marquardt JU, Galle PR, Teufel A (2012). Molecular diagnosis and therapy of hepatocellular carcinoma (HCC): An emerging field for advanced technologies. Journal of Hepatology.

[13] Wicha MS, Liu SL, Dontu G (2006). Cancer stem cells: An old idea - A paradigm shift. Cancer research.

[14] Yamashita T, Ji JF, Budhu A, Forgues M, Yang W, Wang HY, Jia HL, Ye QH, Qin LX, Wauthier E, Reid LM, Minato H, Honda M (2009). EpCAM-Positive Hepatocellular Carcinoma Cells Are Tumor-Initiating Cells With Stem/Progenitor Cell Features. Gastroenterology.

[15] Yamashita T, Honda M, Nio K, Nakamoto Y, Yamashita T, Takamura H, Tani T, Zen Y, Kaneko S (2010). Oncostatin M Renders Epithelial Cell Adhesion Molecule-Positive Liver Cancer Stem Cells Sensitive to 5-Fluorouracil by Inducing Hepatocytic Differentiation. Cancer research.

[16] Bao B, Ahmad A, Kong DJ, Ali S, Azmi AS, Li YW, Banerjee S, Padhye S, Sarkar FH (2012). Hypoxia Induced Aggressiveness of Prostate Cancer Cells Is Linked with Deregulated Expression of VEGF, IL-6 and miRNAs That Are Attenuated by CDF. PloS one.

[17] Airaksinen MS, Saarma M (2002). The GDNF family: signalling, biological functions and therapeutic value. Nature reviews Neuroscience.

[18] Kang J, Qian PX, Pandey V, Perry JK, Miller LD, Liu ET, Zhu T, Liu DX, Lobie PE (2010). Artemin is estrogen regulated and mediates antiestrogen resistance in mammary carcinoma. Oncogene.

[19] Ding KS, Banerjee A, Tan S, Zhao JS, Zhuang Q, Li R, Qian PX, Liu SL, Wu ZS, Lobie PE, Zhu T (2014). Artemin, a Member of the Glial Cell Line-derived Neurotrophic Factor Family of Ligands, Is HER2-regulated and Mediates Acquired Trastuzumab Resistance by Promoting Cancer Stem Cell-like Behavior in Mammary Carcinoma Cells. Journal of Biological Chemistry.

[20] Banerjee A, Qian P, Wu ZS, Ren X, Steiner M, Bougen NM, Liu S, Liu DX, Zhu T, Lobie PE (2012). Artemin stimulates radio- and chemo-resistance by promoting TWIST1-BCL-2-dependent cancer stem cell-like behavior in mammary carcinoma cells. The Journal of biological chemistry.

[21] Mas VR, Maluf DG, Archer KJ, Yanek K, Kong XR, Kulik L, Freise CE, Olthoff KM, Ghobria RM, McIver P, Fisher R (2009). Genes Involved in Viral Carcinogenesis and Tumor Initiation in Hepatitis C Virus-Induced Hepatocellular Carcinoma. Mol Med.

[22] Ma S, Lee TK, Zheng BJ, Chan KW, Guan XY (2008). CD133^+^ HCC cancer stem cells confer chemoresistance by preferential expression of the Akt/PKB survival pathway. Oncogene.

[23] Hu YF, Smyth GK (2009). ELDA: Extreme limiting dilution analysis for comparing depleted and enriched populations in stem cell and other assays. J Immunol Methods.

[24] Thiery JP (2002). Epithelial-mesenchymal transitions in tumour progression. Nature reviews Cancer.

[25] Dubrovska A, Kim S, Salamone RJ, Walker JR, Maira SM, Garcia-Echeverria C, Schultz PG, Reddy VA (2009). The role of PTEN/Akt/PI3K signaling in the maintenance and viability of prostate cancer stem-like cell populations. Proceedings of the National Academy of Sciences of the United States of America.

[26] Li YQ, Laterra J (2012). Cancer Stem Cells: Distinct Entities or Dynamically Regulated Phenotypes?. Cancer research.

[27] Thiery JP, Acloque H, Huang RY, Nieto MA (2009). Epithelial-mesenchymal transitions in development and disease. Cell.

[28] Kurrey NK, Jalgaonkar SP, Joglekar AV, Ghanate AD, Chaskar PD, Doiphode RY, Bapat SA (2009). Snail and slug mediate radioresistance and chemoresistance by antagonizing p53-mediated apoptosis and acquiring a stem-like phenotype in ovarian cancer cells. Stem cells.

[29] Conley SJ, Gheordunescu E, Kakarala P, Newman B, Korkaya H, Heath AN, Clouthier SG, Wicha MS (2012). Antiangiogenic agents increase breast cancer stem cells via the generation of tumor hypoxia. Proceedings of the National Academy of Sciences of the United States of America.

[30] Forsythe JA, Jiang BH, Iyer NV, Agani F, Leung SW, Koos RD, Semenza GL (1996). Activation of vascular endothelial growth factor gene transcription by hypoxia-inducible factor 1. Mol Cell Biol.

[31] Yuan Y, Hilliard G, Ferguson T, Millhorn DE (2003). Cobalt inhibits the interaction between hypoxia-inducible factor-alpha and von Hippel-Lindau protein by direct binding to hypoxia-inducible factor-alpha. The Journal of biological chemistry.

[32] Semenza GL (2014). Oxygen sensing, hypoxia-inducible factors, and disease pathophysiology. Annual review of pathology.

[33] Loots GG, Ovcharenko I (2004). rVISTA 2. 0: evolutionary analysis of transcription factor binding sites. Nucleic acids research.

[34] Fukuda R, Zhang H, Kim JW, Shimoda L, Dang CV, Semenza GL (2007). HIF-1 regulates cytochrome oxidase subunits to optimize efficiency of respiration in hypoxic cells. Cell.

[35] Romano A, Adriaens M, Kuenen S, Delvoux B, Dunselman G, Evelo C, Groothuis P (2010). Identification of novel ER-alpha target genes in breast cancer cells: gene- and cell-selective co-regulator recruitment at target promoters determines the response to 17beta-estradiol and tamoxifen. Molecular and cellular endocrinology.

[36] Luo J, Manning BD, Cantley LC (2003). Targeting the PI3K-Akt pathway in human cancer: Rationale and promise. Cancer Cell.

[37] Hanahan D, Weinberg RA (2011). Hallmarks of cancer: the next generation. Cell.

[38] Kato N, Ji GJ, Wang Y, Baba M, Hoshida Y, Otsuka M, Taniguchi H, Moriyama M, Dharel N, Goto T, Shao RX, Matsuura T, Ishii K (2005). Large-scale search of single nucleotide polymorphisms for hepatocellular carcinoma susceptibility genes in patients with hepatitis C. Hepatology.

[39] Liu S, Gong J, Morishita A, Nomura T, Miyoshi H, Tani J, Kato K, Yoneyama H, Deguchi A, Mori H, Mimura S, Nomura K, Himoto T (2011). Use of protein array technology to investigate receptor tyrosine kinases activated in hepatocellular carcinoma. Exp Ther Med.

[40] Ranieri G, Gadaleta-Caldarola G, Goffredo V, Patruno R, Mangia A, Rizzo A, Sciorsci RL, Gadaleta CD (2012). Sorafenib (BAY 43-9006) in Hepatocellular Carcinoma Patients: From Discovery to Clinical Development. Curr Med Chem.

[41] Bespalov MM, Sidorova YA, Tumova S, Ahonen-Bishopp A, Magalhaes AC, Kulesskiy E, Paveliev M, Rivera C, Rauvala H, Saarma M (2011). Heparan sulfate proteoglycan syndecan-3 is a novel receptor for GDNF, neurturin, and artemin. J Cell Biol.

[42] Ferrara N, Hillan KJ, Gerber HP, Novotny W (2004). Discovery and development of bevacizumab, an anti-VEGF antibody for treating cancer. Nat Rev Drug Discov.

[43] Honma Y, Araki T, Gianino S, Bruce A, Heuckeroth RO, Johnson EM, Milbrandt J (2002). Artemin is a vascular-derived neurotropic factor for developing sympathetic neurons. Neuron.

[44] Li YL, Gan GP, Zhang HZ, Wu HZ, Li CL, Huang YP, Liu YW, Liu JW (2007). A flavonoid glycoside isolated from Smilax china L. rhizome *in vitro* anticancer effects on human cancer cell lines. J Ethnopharmacol.

[45] Nieto MA (2013). Epithelial Plasticity: A Common Theme in Embryonic and Cancer Cells. Science.

[46] Huber RM, Lucas JM, Gomez-Sarosi LA, Coleman I, Zhao S, Coleman R, Nelson PS (2015). DNA damage induces GDNF secretion in the tumor microenvironment with paracrine effects promoting prostate cancer treatment resistance. Oncotarget.

[47] Banerjee A, Wu ZS, Qian P, Kang J, Pandey V, Liu DX, Zhu T, Lobie PE (2011). ARTEMIN synergizes with TWIST1 to promote metastasis and poor survival outcome in patients with ER negative mammary carcinoma. Breast cancer research.

[48] Tsuji K, Kitamura S, Makino H (2014). Hypoxia-inducible factor 1 alpha regulates branching morphogenesis during kidney development. Biochemical and biophysical research communications.

[49] Nozawa-Suzuki N, Nagasawa H, Ohnishi K, Morishige K (2015). The inhibitory effect of hypoxic cytotoxin on the expansion of cancer stem cells in ovarian cancer. Biochemical and biophysical research communications.

[50] Yuan FJ, Zhou WB, Zou C, Zhang ZY, Hu HS, Dai ZQ, Zhang YS (2010). Expression of Oct4 in HCC and modulation to wnt/beta-catenin and TGF-beta signal pathways. Mol Cell Biochem.

[51] Beck B, Driessens G, Goossens S, Youssef KK, Kuchnio A, Caauwe A, Sotiropoulou PA, Loges S, Lapouge G, Candi A, Mascre G, Drogat B, Dekoninck S (2011). A vascular niche and a VEGF-Nrp1 loop regulate the initiation and stemness of skin tumours. Nature.

[52] Banerjee A, Wu ZS, Qian PX, Kang J, Liu DX, Zhu T, Lobie PE (2012). ARTEMIN Promotes De Novo Angiogenesis in ER Negative Mammary Carcinoma through Activation of TWIST1-VEGF-A Signalling. PloS one.

[53] Kang J, Perry JK, Pandey V, Fielder GC, Mei B, Qian PX, Wu ZS, Zhu T, Liu DX, Lobie PE (2009). Artemin is oncogenic for human mammary carcinoma cells. Oncogene.

[54] Pandey V, Perry JK, Mohankumar KM, Kong XJ, Liu SM, Wu ZS, Mitchell MD, Zhu T, Lobie PE (2008). Autocrine human growth hormone stimulates oncogenicity of endometrial carcinoma cells. Endocrinology.

[55] Kong X, Li G, Yuan Y, He Y, Wu X, Zhang W, Wu Z, Chen T, Wu W, Lobie PE, Zhu T (2012). MicroRNA-7 inhibits epithelial-to-mesenchymal transition and metastasis of breast cancer cells via targeting FAK expression. PloS one.

[56] Zhu T, Starling-Emerald B, Zhang X, Lee KO, Gluckman PD, Mertani HC, Lobie PE (2005). Oncogenic transformation of human mammary epithelial cells by autocrine human growth hormone. Cancer research.

[57] Hu X, Ghisolfi L, Keates AC, Zhang J, Xiang S, Lee DK, Li CJ (2012). Induction of cancer cell stemness by chemotherapy. Cell cycle.

[58] Bosman FT, World Health Organization, International Agency for Research on Cancer (2010). WHO classification of tumours of the digestive system.

[59] Park SI, Zhang J, Phillips KA, Araujo JC, Najjar AM, Volgin AY, Gelovani JG, Kim SJ, Wang Z, Gallick GE (2008). Targeting SRC family kinases inhibits growth and lymph node metastases of prostate cancer in an orthotopic nude mouse model. Cancer research.

