# Supplementary material for: Artemin is hypoxia responsive and promotes oncogenicity and increased tumor initiating capacity in hepatocellular carcinoma
Source: Oncotarget. 2015 Dec 12;7(3):3267–82. doi: 10.18632/oncotarget.6572 (PMC4823105; doi:10.18632/oncotarget.6572)
Supplement: Supplementary file 1 [file oncotarget-07-3267-s001.pdf]

Artemin is hypoxia responsive and promotes oncogenicity and increased tumor initiating capacity in hepatocellular carcinoma

Supplementary Materials

A

		Corresponding HCC tissues	
		Negative	Positive
Adjacent non-tumorous tissues	Negative	4	11
	Positive	3	2

B

Parameter	n	Artermin positive expression, n (%)	p value
Age (years)			
≤ 55	92	54 (58.7)	0.146
> 55	58	27 (46.6)	
Gender			
Male	126	66(52.4)	0.362
Female	24	15(62.5)	
Cirrhosis			
Yes	136	74(54.4)	0.752
No	14	7(50.0)	
HBsAg			
Yes	120	68(56.7)	0.190
No	30	13(43.3)	
Grade			
I	5	3 (60.0)	0.790
II	140	76 (54.3)	
III	5	2 (40.0)	

C

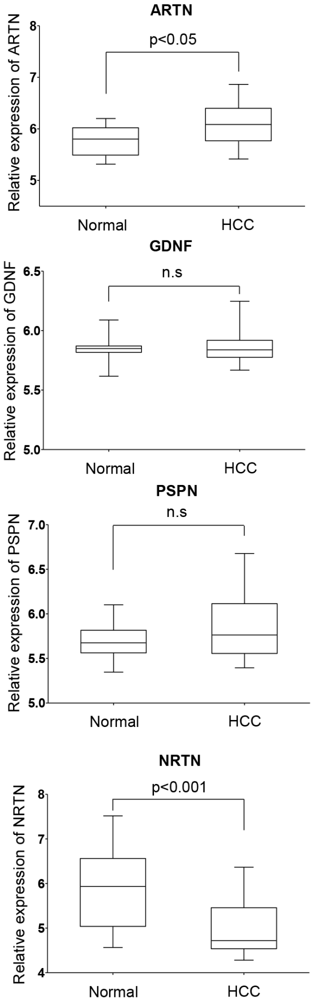

D

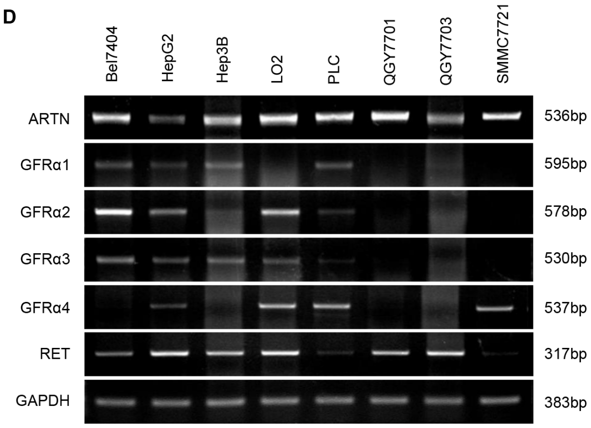

E **F**

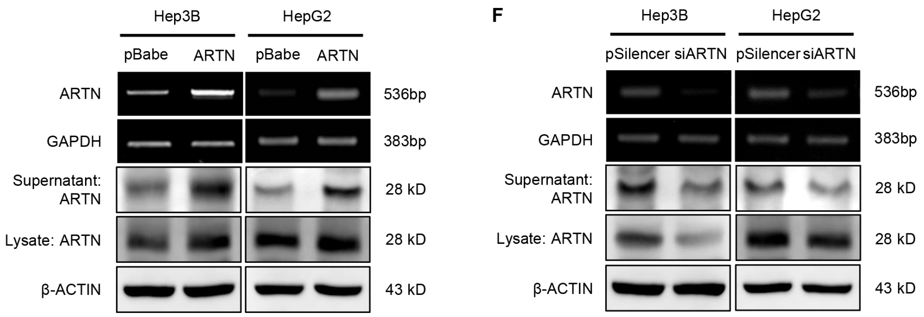

Supplementary Figure S1: ARTN expression patterns in HCC specimens, HCC cell lines and LO2 cells. (**A**) IHC analysis of ARTN expression in human HCC specimens and paired adjacent non-tumorous liver tissues (paired Student *t* test). (**B**) The relationship between ARTN and other clinicopathological characteristics in HCC patients. (**C**) The mRNA expressions of all GDNF family members in HCC samples compared with normal liver tissues based on a published HCC mRNA array dataset. (**D**) The expression of ARTN, GFRα1-4 and RET mRNA in HCC cell lines, determined by RT-PCR. (**E–F**) Verification of forced and knockdown expression of ARTN in Hep3B and HepG2 cell lines by RT-PCR and Western blot ($\chi 2$ test).

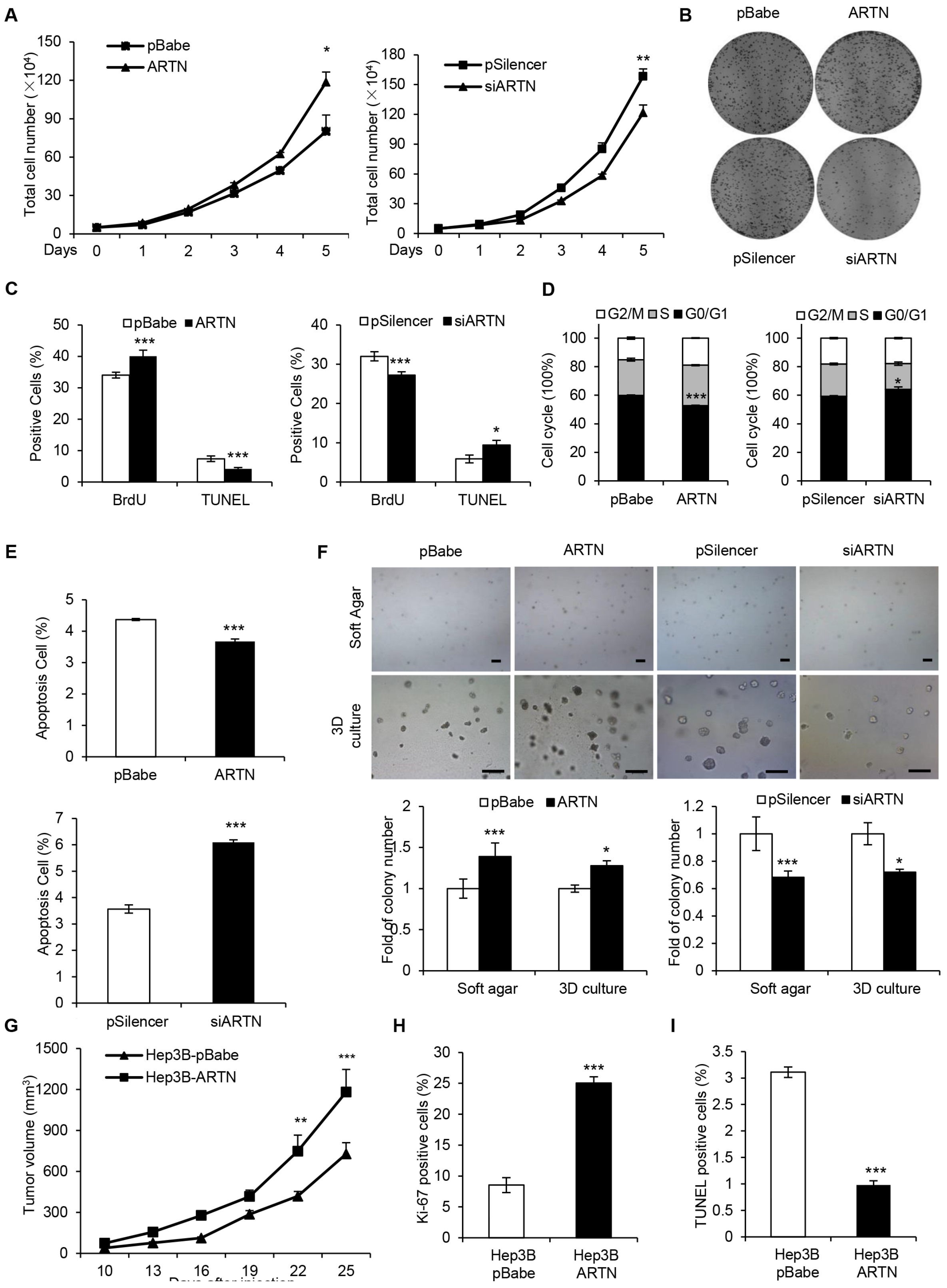

Supplementary Figure S2: ARTN promotes oncogenicity of HepG2 cells *in vitro*. (**A**) Proliferation of HepG2 cells was determined by total cell number counting. (**B**) Foci formation assay of HepG2 cells in monolayer culture. (**C**) The percentages of BrdU and TUNEL positive HepG2 cells. (**D**) Cell cycle analysis. The HepG2 cells were stained with PI and then detected by flow cytometry. The percentages of cells in three phases are shown in histogram. (**E**) Apoptosis cells were detected by Annexin V and PI staining with flow cytometry. (**F**) Soft agar (Upper panels, 40×) and 3D Matrigel (Lower panels, 100×) colony formation of HepG2 cells. Colony numbers were counted and are shown in histogram. (**G**) Tumor growth curve of Hep3B-pBabe and Hep3B-ARTN cells injected into the flank of male nude mice. (**H–I**) Tumor cell proliferation and apoptosis were examined by Ki-67 and TUNEL staining, respectively. Mean ± SD, $n = 3$, $^*P < 0.05$; $^{**}P < 0.01$; $^{***}P < 0.001$ (Student t test).

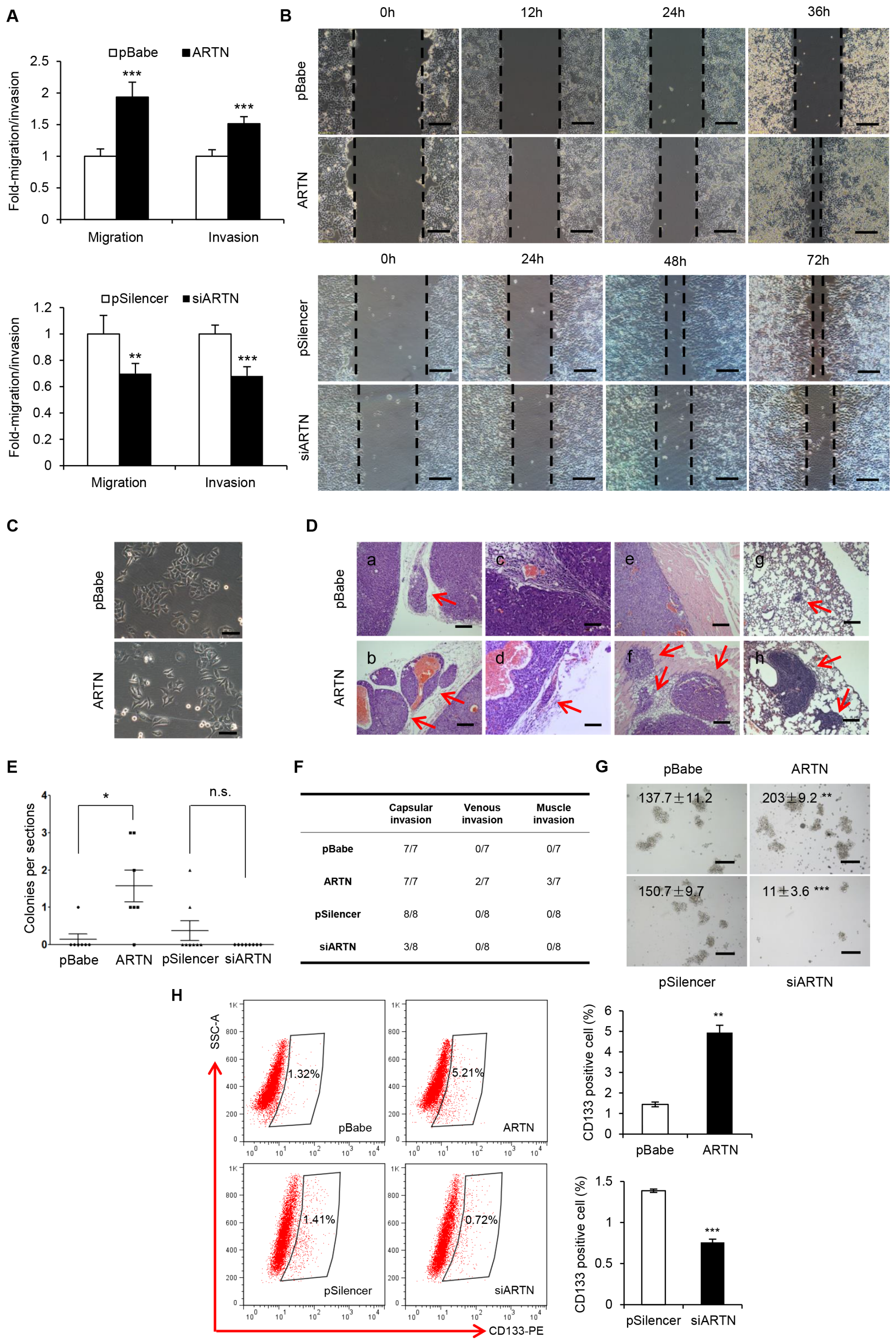

	Capsular invasion	Venous invasion	Muscle invasion
pBabe	7/7	0/7	0/7
ARTN	7/7	2/7	3/7
pSilencer	8/8	0/8	0/8
siARTN	3/8	0/8	0/8

Supplementary Figure S3: ARTN regulates the metastatic capacity and CSC properties of HepG2 cells *in vitro*. (**A**) Transwell migration and invasion assay of HepG2 cells. (**B**) Wound healing assay of HepG2-ARTN and HepG2-siARTN cells compared with their respective control cells. Magnification, 100×. (**C**) Morphology of HepG2-pBabe and HepG2-ARTN cells. Representative pictures were taken using phase-contrast microscopy at 200× magnification. (**D**) H & E staining of tumors and lungs from mice xenograft model. Arrows indicated invasion of capsular (a, b), venous (c, d), muscles (f) and lung metastasis foci (g, h). (**E**) Numbers of lung micrometastases per section in individual mice (each data point represented a different mouse). (**F**) The quantification of the mice showed invasive phenotypes in each group. (**G**) Tumorsphere formation assays of HepG2 cells. Representative images of tumorspheres generated by HepG2 cells were captured by microscope at 100× magnification. (**H**) CD133 positive population in HepG2 cells. Results were shown in histogram. Mean ± SD, $n = 3$, $*P < 0.05$; $**P < 0.01$; $***P < 0.001$ (Student t test).

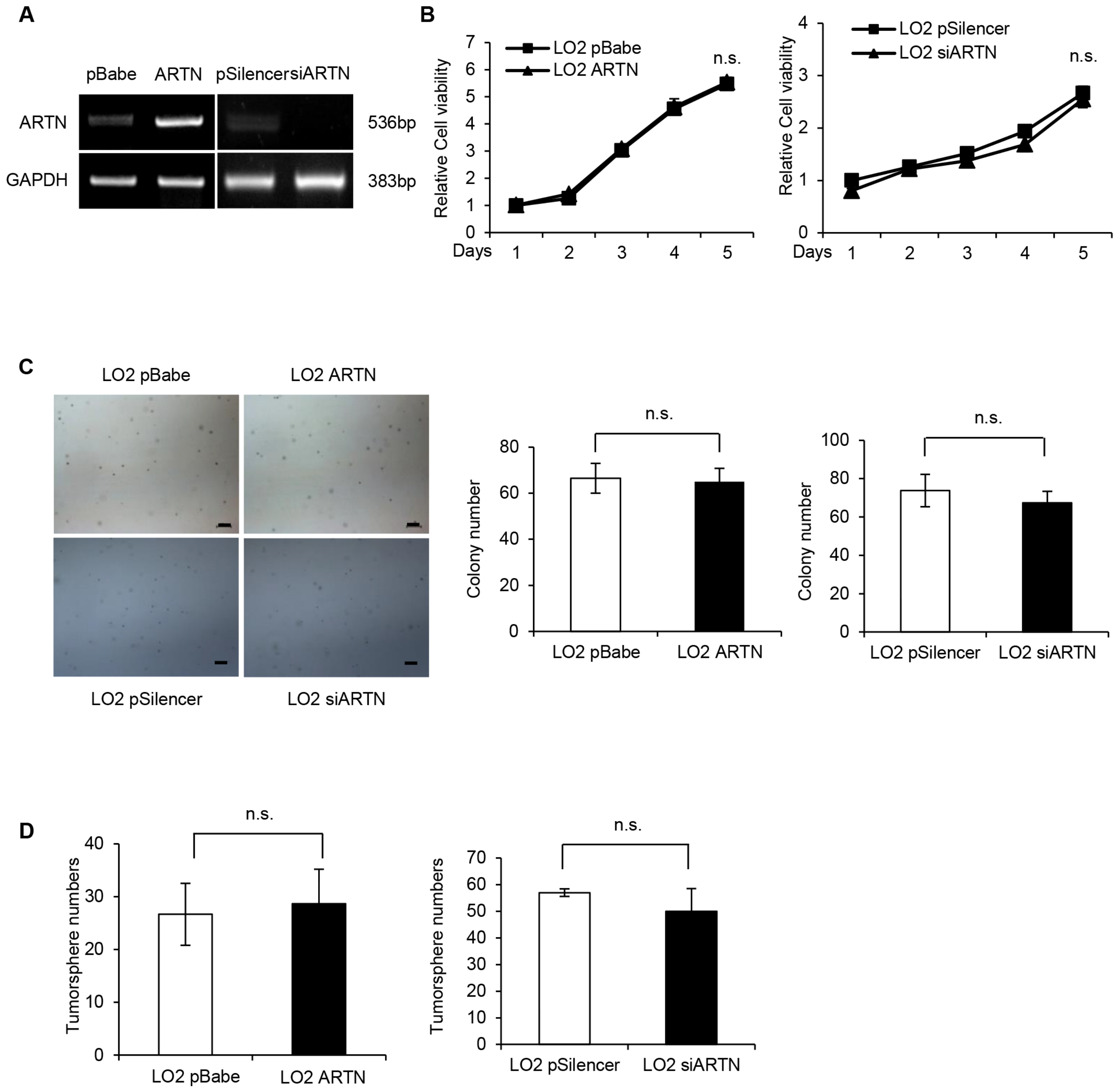

Supplementary Figure S4: ARTN doesn't affect the viability, motility and CSC properties of normal liver cells. (**A**) Establishment of ARTN overexpression and knockdown in LO2 cells was verified by RT-PCR. (**B**) Relative cell viability of LO2-ARTN and LO2-siARTN cells compared with their respective control cells. Cells were incubated with CCK8 and then detected at the wavelength of 450 nm. (**C**) Soft agar assay of LO2 cells. Representative images of colonies formed by LO2 cells were taken at 40× magnification. The results were shown in histogram. (**D**) Tumorsphere formation of LO2 cells. Mean ± SD, $n = 3$, n.s., not significant (Student t test).

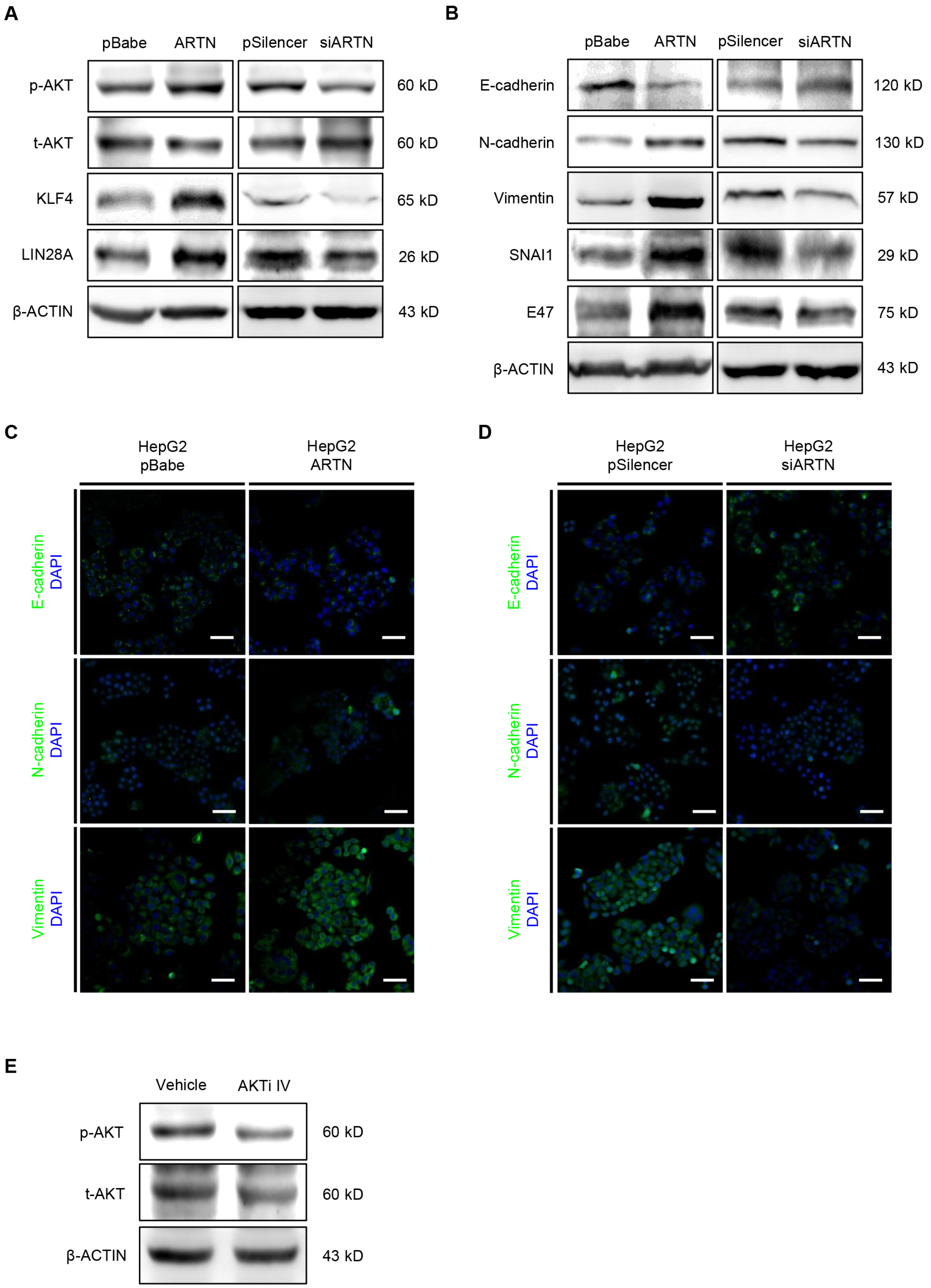

Supplementary Figure S5: ARTN modulates CSC and EMT via regulation of several important factors. (**A**) Western blot analysis of p-AKT and important transcription factors (KLF4, LIN28A) of CSC pathway in HepG2 cells. (**B**) Western blot analysis of EMT markers and inducers in HepG2 cells. (**C–D**) Immunofluorescene staining of E-cadherin, N-cadherin and Vimentin in HepG2 cells. Representative pictures were taken using confocal microscopy at 200 × magnification. (**E**) The p-AKT level was detected after treatment of AKT inhibitor IV (AKTi IV) for 24 hours.

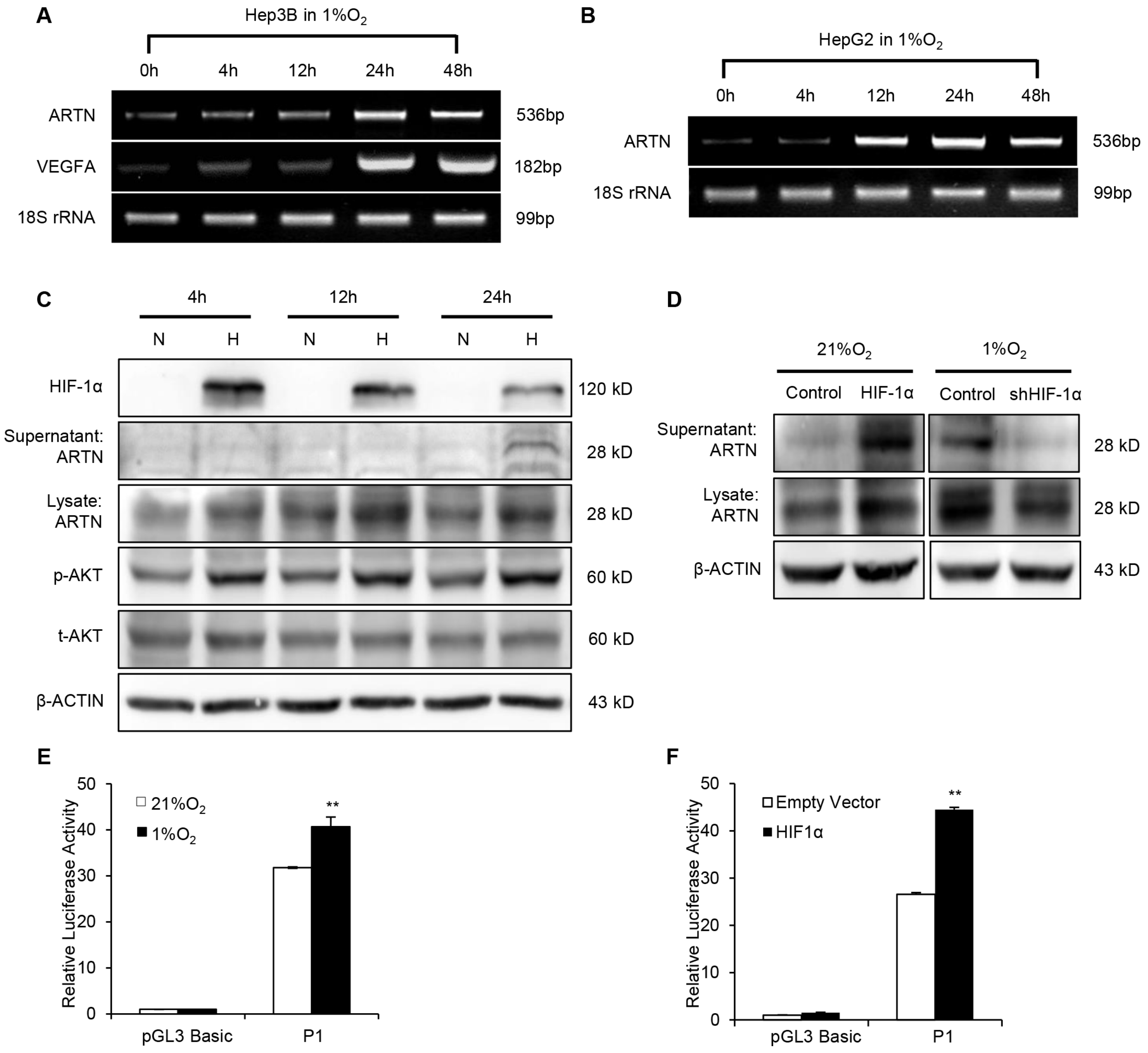

Supplementary Figure S6: ARTN is induced by hypoxic condition in HCC cells. (**A**) Time-course analysis of ARTN mRNA under hypoxia condition in Hep3B cells. The levels of ARTN mRNA were determined by RT-PCR. VEGFA mRNA was used as a positive control. (**B**) Time-course analysis of ARTN mRNA under hypoxia condition in HepG2 cells. (**C**) Time-course analysis of HIF-1α, ARTN and p-AKT protein in HepG2 cells. Cells were incubated under normoxia or hypoxia for every indicated time. The protein levels were determined by Western blot. N, 21% O_2; H, 1% O_2. (**D**) The expression of ARTN was related with HIF-1α level in HepG2 cells, determined by Western blot. In normoxia, HepG2 cells were transiently transfected with HIF-1α cDNA or the control construct, or HIF-1α was knockdown by shRNA plasmid under hypoxia for 48 hours. (**E**) Luciferase activity of promoter (P1). HepG2 cells were transfected with the promoter (P1) constructs and then incubated in normoxic (21% O_2) or hypoxic (1% O_2) conditions. (**F**) Analysis of promoter (P1) activity in HepG2 cells after transfection of HIF-1α cDNA or the control construct under normoxia. Mean ± SD, $n = 3$, $^{**}P < 0.05$ (Student t test).

Supplementary Table S1: Sequences of the oligonucleotides for RT-PCR analysis, shRNA, plasmid construction and ChIP assays

**Primers for RT-PCR**	**Sense Strand (5′-3′)**	**Antisense Strand (5′-3′)**
ARTN	TGAGCAGCGTCGCAGAGG	GAGGCGGTCCACGGTTCT
GFRα1	TTGCAGGACTCCTGCAAGACG	GACCACAGCTTGGAGGAGCAG
GFRα2	CAGCTGCTCCTATGAGGACAAG	CTGGGATGATATTTGTCGTGAGC
GFRα3	CTGCTCACTTTCTTCGAGAAGG	CAGGGTTTTCATTCTGGTGTGC
GFRα4	ATGGTGCCATTCAGGCCTTTGC	ATGGTCTCTGACCTGCTCTAGG
RET	CGTGAAGAGGAGCCAGGGTC	TAACCATCATCTTCTCCAGGTCT
GAPDH	TCCCATCACCATCTTCCAGG	CCATCACGCCACAGTTTCC
VEGFA	ACGGACAGACAGACAGACACC	GAACAGCCCAGAAGTTGGAC
18S rRNA	CGGCGACGACCCATTCGAAC	GAATCGAACCCTGATTCCCCGTC
**Primers for plasmid construction**	**Sense Strand (5′-3′)**	**Antisense Strand (5′-3′)**
ARTN cloning primer	CGGATCCATGGAACTTGGACTTGGAGGCC	AGAATTCTCAGCCCAGGCAGCCGCAGG
siARTN cloning primer	AACAGCACCTGGAGAACCG	
ARTN promoter P1 cloning primer	ATAAGCTAGCAACCCTCCCATGGTCCCCAGTG	CGTAAGCTTGGCAAGTGGGCTGGCTTACCTC
ARTN promoter P2 cloning primer	CCGAGCTAGCTTTAGCCTGGACCAAAGGGAAG	CGTAAGCTTGGGAGGGTTTTGAGCAGAATGG
ARTN promoter P1 HIF-1α Mut A primer	GGACCCCCAAATCTCAGCAGTCAGCCGCCC	GGGCGGCTGACTGCTGAGATTTGGGGGTCC
ARTN promoter P1 HIF-1α Mut B primer	CAGCAGTCAGCCGCCGGACCGGCTTACCCC	GGGGTAAGCCGGTCCGGCGGCTGACTGCTG
sh Scr	CCTAAGGTTAAGTCGCCCTCGCTCGAGCGAGGGCGACTTAACCTTAGG	
sh HIF-1α	CCGGCCAGTTATGATTGTGAAGTTACTCGAGTAACTTCACAATCATAACTGGTTTTT	
**Primers for ChIP assay**	**Sense Strand (5′-3′)**	**Antisense Strand (5′-3′)**
HIF-1α Site A and B Primers	GAAGAATCGGGTGGAGCA	GGAGCGAGGGGTAAGCC
NC site Primer	ATGGTTGCCACTGGGGATCT	TGCCAAAGCCTAGGGGAAGA

Supplementary Table S2: List of proteins tested by antibodies and characteristics of the corresponding antibodies used

Protein	Assay	Antibody	Origin	Dilution	Incubation period
ARTN	WB	gpab	#sc-9330, Santa Cruz	1:1000	overnight
ARTN	IHC	gpab	#sc-9330, Santa Cruz	1:200	overnight
CD133	FACS	mmab	#130080801, Miltenyi Biotec	0.5 μg	10 min
ACTIN	WB	mmab	#M20010, Abmart	1:5000	overnight
Phospho-AKT	WB	rpab	#CST9271, Cell Signaling	1:1000	overnight
Total-AKT	WB	mmab	#sc-5298, Santa Cruz	1:1000	overnight
KLF4	WB	mmab	#AM2725a, Abgent	1:1000	overnight
LIN28A	WB	rpab	#3978, Cell Signaling	1:1000	overnight
E-Cadherin	WB	mmab	#610181, BD	1:5000	overnight
E-Cadherin	IF	mmab	#610181, BD	1:400	overnight
N-Cadherin	WB	mmab	#610920, BD	1:5000	overnight
N-Cadherin	IF	mmab	#610920, BD	1:400	overnight
VIMENTIN	WB	mmab	#550513, BD	1:5000	overnight
VIMENTIN	IF	mmab	#550513, BD	1:400	overnight
SNAIl	WB	rpab	#sc-28199, Santa Cruz	1:1000	overnight
E47	WB	mmab	#554199, BD	1:5000	overnight
HIF-1α	WB	mmab	#610958, BD	1:5000	overnight
HIF-1α	IHC	mmab	#610958, BD	1:400	2 hour
HIF-1α	CHIP	rpab	# NB100-134, Novus	10μg	overnight

Abbreviations: WB, Western blot; FACS, flow cytometry; ChIP, chromatin immunoprecipitation; IHC, immunohistochemistry; IF, immunofluorescence; mmab, mouse monoclonal antibody; rpab, rabbit polyclonal antibody; gpab, goat polyclonal antibody.
